# Efficient biodegradation and upcycling of polyethylene terephthalate mediated by cell-factories

**DOI:** 10.3389/fmicb.2025.1599470

**Published:** 2025-07-02

**Authors:** Fei Liu, Tao Wang, Xiao-huan Liu, Na Xu, Xing-li Pan

**Affiliations:** ^1^School of Life Sciences, Jining Medical University, Jining, China; ^2^School of Pharmacy/School of Modern Chinese Medicine Industry, Chengdu University of Traditional Chinese Medicine, Chengdu, China

**Keywords:** polyethylene terephthalate, microbial degradation, PET recycling, metabolic engineering, synthetic biology, cell factory

## Abstract

The pervasive accumulation of polyethylene terephthalate (PET) waste has emerged as a critical ecological crisis, which is mainly driven by its recalcitrance to natural degradation and widespread contamination of terrestrial and aquatic ecosystems. In response to this challenge, microbial-mediated PET biodegradation has garnered significant scientific attentions as a sustainable remediation strategy, harnessing the enzymatic cascades of specialized microorganisms to depolymerize PET into bio-assimilable monomers such as terephthalic acid (TPA) and ethylene glycol (EG). In this review, we summarize the extracellular process of PET biodegradation, including microbial attachment, colonization, and direct depolymerization, as well as the metabolic pathways of PET monomers. Strategies for developing PET-degrading chassis cells are also discussed, such as cell surface display, metabolic pathway optimization, and rational design of enzyme-PET interfaces. Microbial-enzyme consortia and molecular engineering of photosynthetic microorganisms also contribute to PET degradation. Although significant progress has been made, challenges remain in enzyme stability, metabolic bottlenecks, industrial scalability, and environmental adaptation. Overall, microbial and enzymatic strategies show great potentials in addressing PET pollution, and future interdisciplinary efforts are needed to overcome these challenges and achieve a sustainable circular plastic economy.

## 1 Introduction

Polyethylene terephthalate (PET), renowned for its excellent physical and chemical properties, is one of the most extensively used synthetic polymers globally. Owing to its remarkable durability, versatility, and cost-effectiveness, PET has found extensive application across a diverse range of industries, including packaging, textiles, and electronics. However, the widespread use of PET has also led to a significant accumulation of plastic waste in the environment (MacLeod et al., 2021; Santos et al., 2021). PET is highly resistant to natural degradation processes, with a long half-life that can span hundreds of years in landfills, oceans, and other ecosystems (Chu et al., 2022; Zhu et al., 2024). This has given rise to severe environmental concerns, including soil and water pollution, harm to wildlife, and negative impacts on human health (Preda et al., 2024; Li Y. et al., 2024).

**FIGURE 1:**
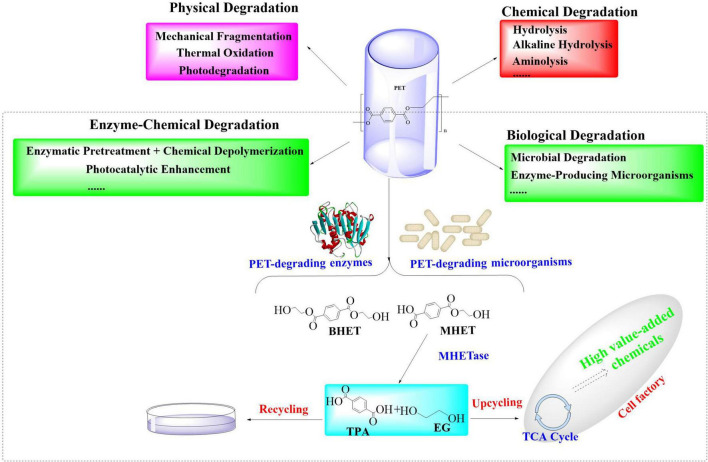
Summary of the established PET-degradation pathways.

Current PET recycling strategies include mechanical recycling and energy-intensive chemical methods (e.g., glycolysis and hydrolysis) (Cao et al., 2022; Mahler et al., 2025; Ragaert et al., 2017), which usually require high temperatures (>200°C) and generate toxic byproducts. While thermal incineration would recover energy, it might emit CO_2_ and other hazardous pollutants. These approaches would also struggle with scalability, cost, and environmental trade-offs. Additionally, biological PET-degradation, especially the microbial-mediated biodegradation, has emerged as a promising and sustainable solution to address this ecological crisis (Figure 1; Mudondo et al., 2023; Bian et al., 2024). Certain microorganisms could employ endogenous enzymatic cascades to depolymerize PET into monomers (Yip et al., 2024; Yoshida et al., 2021; Barone et al., 2024), which offers a sustainable pathway to mitigate PET pollution. Therefore, understanding the metabolic pathways involved in PET biodegradation is fundamental for harnessing the full potentials of the PET-degrading microorganisms. However, the efficiency of native microbial systems usually remains constrained, such as by suboptimal enzyme activity, substrate accessibility, and inhibitory byproduct accumulation. To address these limitations, enzyme molecular engineering has emerged as a promising approach to enhance PET depolymerization by optimizing the catalytic efficiency of these enzymes (Arnal et al., 2023; Liu F. et al., 2023). Furthermore, the use of synthetic biology and metabolic engineering has further facilitated these processes, leading to significant advancements in PET biodegradation (Bian et al., 2024; Diao et al., 2023). Examples include directed evolution (Shi et al., 2023), dual-enzyme systems (Aer et al., 2024b), surface-display technologies (Xue et al., 2024), and microbial consortia (Salinas et al., 2025), which have demonstrated significant potential in overcoming critical barriers such as intermediate metabolite accumulation and hydrophobic substrate interactions.

Cell factories, the engineered microorganisms with optimized metabolic capabilities, play a crucial role in multiple key aspects (Cho et al., 2022; Qiu et al., 2023; Hussain et al., 2022; Zuo et al., 2024), such as the realm of biotechnology and industrial production, the pharmaceutical industry, the field of environmental remediation, the production of fine chemicals and industrial enzymes. Through metabolic engineering and synthetic biology approaches, the genetic makeup of chassis cells could be modified and optimized, which ensures that the cellular metabolic pathways (metabolic flux) are finely tuned. Equipped with optimal PET hydrolases and optimized metabolic pathways, the engineered cell factories would demonstrate enhanced degradation rates and sustainable utilization of the degradation products (Moog et al., 2021; Dissanayake and Jayakody, 2021; Valenzuela-Ortega et al., 2023; Schneier et al., 2024). Especially, once PET is broken down, the resulting monomers and other small-molecule products would be channeled into the well-designed biosynthetic pathways within the cells (Mubayi et al., 2024; Yang et al., 2024). For instance, the monomers can be used as building blocks for the synthesis of other high-value chemicals (Diao et al., 2024; You et al., 2023), such as specialty solvents, pharmaceutical precursors, or bio-based polymers. This not only reduces the reliance on fossil-based feedstocks but also minimizes the environmental impact associated with conventional plastic production processes. The circular plastic economy seeks to eradicate waste generation through perpetual material recycling into novel products, achieved by implementing innovative strategies for designing efficient metabolic pathways to facilitate monomer conversion. The closed-loop system created by these biological systems ensures that materials are recycled and reused, reducing waste generation and promoting environmental sustainability (Rani et al., 2024; Arijeniwa et al., 2024).

This review analyzes the current state-of-the-art in microbial-mediated PET biodegradation. It is mainly focused on the key enzymes and the metabolic pathways involved in PET degradation, and the strategies for engineering cell factories with enhanced PET degradation efficiency. Additionally, it also explores the upcycling of PET degradation products into valuable chemicals to offer a sustainable approach for plastic waste management. By presenting a detailed overview of these aspects, we hope to provide valuable insights for researchers in this field, and facilitate the development of more effective and environmentally friendly solutions for PET biodegradation.

## 2 Microbial-mediated degradation of polyethylene terephthalate

### 2.1 The extracellular process of PET bio-degradation

The process of microbial-mediated degradation of plastic polymers usually encompasses several key steps (Jaiswal et al., 2019), including biodeterioration, bio-fragmentation, assimilation, and mineralization. To achieve optimal microbial utilization for PET biodegradation, a systematic elucidation of metabolic mechanisms must integrate genomic engineering, enzymatic optimization, and ecological synergy. This will guide strategies to engineer microbial platforms with dual catalytic and PET upcycling capabilities, which paves the way for innovative plastic-to-value conversion systems.

#### 2.1.1 The process of microbial attachment and colonization

During the process of microbial-mediated PET biodegradation, microorganisms must firstly be able to adhere to and proliferate on the surface of PET plastic, which is achieved through a combination of biological and physicochemical mechanisms. For example, specific species within the genus *Bacillus* and *Pseudomonas* exhibit an affinity for polymers like PET. Moreover, they usually possess the ability to form biofilms on the surfaces of PET materials, which would allow them to persist and colonize surfaces (Skariyachan et al., 2017; Sivan et al., 2006). This is a crucial step in the biodegradation process. In *Bacillus* species, the formation of biofilms is usually regulated by the two-component signal transduction systems (TCSs) (Ducret et al., 2022), which are typically composed of a sensor histidine kinase (HK) and a response regulator (RR).

Furthermore, it was found that several factors are capable of regulating the surface-attachment process, which can influence how microorganisms or the enzymes interact with surfaces. The hydrophobins (HPs), a kind of small secreted surface-active proteins, could also mediate fungal attachment to hydrophobic surfaces like PET (Terauchi et al., 2022; Sánchez, 2020). In light of this, HPs may serve as effective substrates to enhance microbial adhesion, particularly for strains expressing surface-displayed PET-degrading enzymes, thus significantly improving enzymatic degradation efficiency. Moreover, the implementation of chimeric anchor motifs (e.g., *Bacillus subtilis*-derived LCI modules) also enables precision immobilization of enzymatic machineries on PET matrices through affinity-driven molecular recognition (Liu Y. et al., 2024). This would establish a bioengineered interface that amplifies substrate-enzyme dynamics and potentiates depolymerization within catalytic microenvironments. Additionally, exopolysaccharides also play roles in the attachment, biofilm adhesion and biodeterioration of plastic polymers (A et al., 2020). Manipulating the levels of the second messenger molecule (cyclic-di-GMP), which regulates biofilm formation, has been demonstrated to significantly enhance the plastic degradation rates (Howard and McCarthy, 2023). The introduction of diguanylate cyclases (DGCs) into *Escherichia coli* strains expressing PET-degrading enzymes resulted in a notable increase in polyester degradation, which highlights that adjusting biofilm levels can boost enzyme efficacy.

#### 2.1.2 The process of direct PET-depolymerization

After adhering to the surface and establishing colonization, microorganisms are likely to actively secrete extracellular PET-degrading enzymes specifically bound to PET surfaces, thereby facilitating the biodegradation process. In this stage, two different catalysts would be produced (Table 1) (Kawai et al., 2019): the surface modification enzymes (associated with enzymatic modification of PET to increase the surface hydrophilicity) and the PET-degrading enzymes (capable of directly breaking the ester bonds within the PET polymer releasing monomeric products). PET hydrolases and surface modification enzymes play distinct roles in PET biodegradation. PET hydrolases (e.g., *Is*PETase) directly cleave ester bonds in PET, depolymerizing it into soluble monomers like terephthalic acid (TPA), ethylene glycol (EG), mono(2-hydroxyethyl) terephthalate (MHET), and Bis(2-hydroxyethyl) terephthalate (BHET). These enzymes usually exhibit catalytic triads (e.g., S160-D206-H237 in *Is*PETase) and structural adaptations (e.g., disulfide bridges) for substrate binding and hydrolysis. In contrast, surface modification enzymes (e.g., lipases and carboxylesterases) enhance PET hydrophilicity by weakening intermolecular forces, reducing mechanical strength, and increasing water affinity. While hydrolases target chemical bonds, surface modifiers precondition the polymer physically, enabling efficient depolymerization by hydrolases. Lipases, carboxylesterases, and most cutinases exhibit surface modification capabilities that reduce PET strength by weakening intermolecular forces. This would compromise mechanical properties while accelerating degradation susceptibility. For example, lipase from *Aspergillus oryzae* was used as a catalyst for enzymatic modification of PET fabric combined with polyethylene glycol (PEG), which showed that the water contact angle (WCA) of the treated fabric was significantly reduced from 129.3° to 74.9°, while the moisture regain increased to 3.52% (Liu W. et al., 2024). The lipase from *Thermomyces lanuginosus* was also successfully used for the biodegradation of PET fabrics/films combined with cutinases from *Thermobifida fusca* and *Fusarium solani* (Eberl et al., 2009). In addition to surface-modification, the lipase B from *Moesziomyces antarcticus* (CALB) was also found to be capable of efficiently converting BHET to MHET and subsequently MHET to TPA and EG (Carniel et al., 2017).

**TABLE 1:** The enzymes utilized for PET degradation.

		Enzymes	Source	References
Carboxylic ester hydrolase-derived PET hydrolases	Lipases	BsEstB	*Bacillus subtilis* 4P3-11	Ribitsch et al., 2011
		CALB	*Candida antarctica*	Carniel et al., 2017
	Cutinases (α/β hydrolase family)	Cut190	*Saccharomonospora viridis* AHK190	Kawai et al., 2014
		FsC	*Fusarium solani pisi*	Hellesnes et al., 2023
		HiC	*Humicola insolens*	Romano et al., 2023
		LCC	Leaf-cutinase branch compost metagenome	Sulaiman et al., 2012
		Thc_Cut1, Thc_Cut2	*Thermobifida cellulosilytica* DSM44535	Herrero Acero et al., 2011
		Thf42_Cut1	*Thermobifida fusca* DSM44342	Herrero Acero et al., 2011
		AdCut	*Acidovorax delafieldii*	Clark et al., 2024
	Polyesterase	MoPE	Antarctic bacterium *Moraxella* sp.	Nikolaivits et al., 2022
		jmPE13, jmPE14	*Pseudomonas* sp. JM16B3	Zhou et al., 2024
		BgP	*Brachybacterium ginsengisoli* B129SM11	Carr et al., 2022
		PsP1, PsP2	*Pseudomonas stutzeri*	Howard et al., 2023
	Esterase	TfH, BTA2, Tfu_0882, TfCut1, and TfCut2	*Thermobifida fusca*	Then et al., 2015
		Thh_Est	*Thermobifida halotolerans*	Ribitsch et al., 2012
PETsae	*Is*PETase		*Ideonella sakaiensis* 201-F6	Yoshida et al., 2016
	*Dm*PETase		*Deinococcus maricopensis*	Makryniotis et al., 2023
	SM14est		*Streptomyces* sp. SM14	Almeida et al., 2019
	Ple629		A marine microbial consortium	Meyer-Cifuentes and Öztürk, 2021
MHETase	IsMHETase		*Ideonella sakaiensis* 201-F6	Yoshida et al., 2016
	KL-MHETaseI171K/G130L		/	Zhang J. et al., 2023
	Mle046, Mle800, Mle267, Mle288		A marine microbial consortium	Meyer-Cifuentes and Öztürk, 2021; Meyer-Cifuentes et al., 2020
	PsM1		*Pseudomonas stutzeri*	Howard et al., 2023

On the contrary, a limited number of cutinases and the PETase have been recognized as the PET-degrading enzymes (or named as PET hydrolases), which are capable of breaking down PET directly into smaller, water-soluble molecules. The cutinase, such as *T. fusca* cutinase (TfCut2) (Mrigwani et al., 2023), *F. solani pisi* cutinase (FsC) (Hellesnes et al., 2023), leaf-branch compost cutinase (LCC) (Sulaiman et al., 2012) or their mutants (Tournier et al., 2020; Hong et al., 2023), have all been successfully applied for PET hydrolysis. For instance, the thermostable variant of LCC (LCC*^ICCG^*) demonstrated exceptional catalytic efficiency, and it was able to degrade 90% of the pretreated PET bottles within 10 h (Tournier et al., 2020). In addition, the recently identified mesophilic *Is*PETase and MHETase from *Ideonella sakaiensis* 201-F6 have drawn great attentions (Yoshida et al., 2016), which could catalyze the biodegradation of low-crystallinity (1.9%) PET film at room temperature (30°C). Though *Is*PETase displays a lower PET-degrading activity than cutinase or cutinase-like PET-degrading enzymes, it provides a more environmentally friendly and energy-saving alternative for PET recycling. On this basis, various PETase mutants have been developed with better catalytic performance, and increased thermostability such as HotPETase (Bell et al., 2022), DuraPETase (Cui et al., 2021), Fast-PETase (Lu et al., 2022), TS-PETase (Zhong-Johnson et al., 2021), ThermoPETase (Francis et al., 2019) and the fused protein with the carbohydrate-binding module (CBM) (Dai et al., 2021; Wang T. et al., 2024) or hydrophobin (HFBI) (Chen et al., 2022).

It is important to highlight that the intermediates and final products of PET degradation (e.g., BHET and MHET) have been identified as competitive inhibitors of PET hydrolases (Barth et al., 2015a). Especially, during the hydrolysis of PET, MHET tends to accumulate to a greater extent than BHET, moreover MHET exhibits a strong affinity for the catalytic site of PET hydrolases (Barth et al., 2015b). Therefore, the accumulation of MHET would significantly impede the efficiency of enzymatic PET depolymerization. Consequently, the ongoing removal of soluble metabolites of PET-degradation is essential for overcoming competitive inhibition (Kushwaha et al., 2022).

Thermostable cutinases like LCC*^ICCG^* exhibit exceptional thermostability and display improved catalytic efficiency on semi-crystalline PET, which made them ideal for the industrial-scale processes requiring high-temperature pretreatment. In contrast, mesophilic PETase (e.g., *Is*PETase) are found to be capable of being operated at ambient temperatures (30°C) but suffered from low activity and poor stability, which might limit its industrial viability. However, its energy-saving potential makes it environmentally advantageous for *in situ* remediation (Mrigwani et al., 2023). Advanced PETase mutants (e.g., DuraPETase and Fast-PETase) now enable gap closure through optimized mesophilic functionality (35°C) coupled with 4.2-fold increased degradation rates compared to wild-type enzymes.

### 2.2 Microbial-mediated biodegradation pathway of PET

**FIGURE 2:**
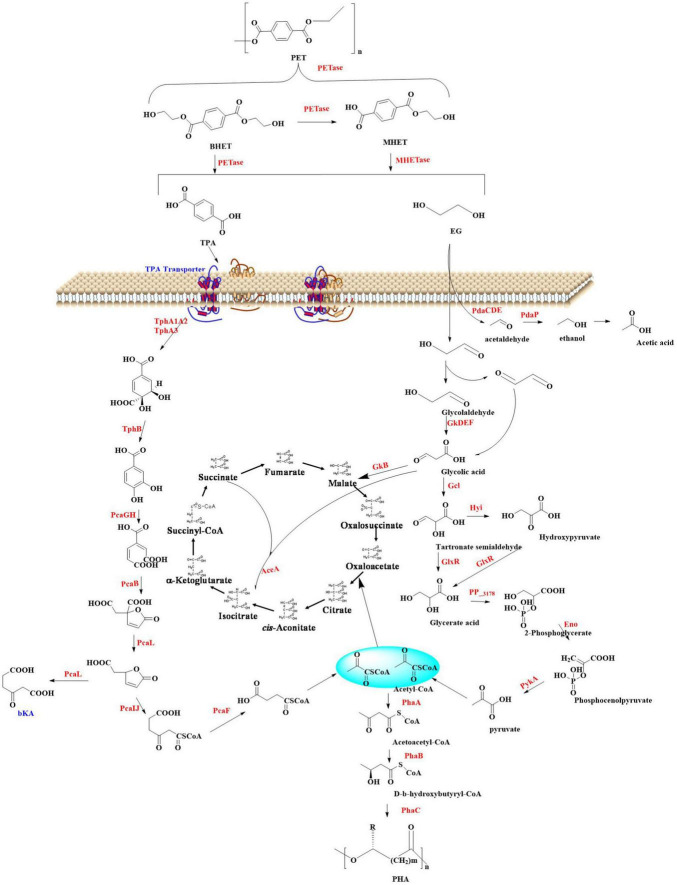
Polyethylene terephthalate metabolic pathways and its conversion into high value chemicals.

The extracellular enzymatic hydrolysis of PET begins with the cleavage of ester bonds through enzymatic action, yielding aromatic intermediates such as TPA, BHET, MHET, and EG. This initial depolymerization phase constitutes the kinetic bottleneck in PET decomposition. The released depolymerization products (primarily TPA and EG) undergo sequential internalization and catabolism through microbial peripheral metabolic pathways (Kushwaha et al., 2022; Gao et al., 2022; Qi et al., 2021b). This process drives cellular proliferation by redirecting carbon flux within metabolic networks, ultimately sustaining biomass synthesis through optimized carbon allocation strategies. Ultimately, biodegradation achieves complete mineralization through coordinated metabolic pathways. In this process, organic substrates are fully oxidized into inorganic end-products (CO_2_ and H_2_O) *via* the tricarboxylic acid (TCA) cycle and associated intracellular redox mechanisms. This process could be illustrated by Figure 2 (Kushwaha et al., 2022; Gao et al., 2022; Qi et al., 2021b).

#### 2.2.1 The metabolic pathways of TPA

Terephthalic acid is taken up by microorganisms through specific transporters such as the MucK in *Acinetobacter baylyi* ADP1 (Pardo et al., 2020). Once inside the cell, TPA would be catabolized *via* the protocatechuate (PCA) 4,5-cleavage pathway (Sasoh et al., 2006; Fukuhara et al., 2008). In this process, genes responsible for TPA degradation are situated within two nearly identical clusters: *tphRICIA2IA3IBIA1I* and *tphRIICIIA2IIA3IIBIIA1II*. The genes (*tphR*, *tphC*, *tphA2*, *tphA3*, *tphB*, and *tphA1*) are anticipated to encode for the IclR-type transcriptional regulator, the TPA binding receptor, the large subunit of the oxygenase component of TPA 1,2-dioxygenase (TPADO), the small subunit of the oxygenase component of TPADO, a 1,2-dihydroxy-3,5-cyclohexadiene-1,4-dicarboxylate dehydrogenase (DCDDH), and a reductase component of TPADO, respectively. The TPADO is capable of converting TPA into 1,2-dihydroxy-3,5-cyclohexadiene-1,4-dicarboxylate, which would be further converted into protocatechuic acid (PCA) catalyzed by DCDDH. The PCA is then ring-cleaved by PCA 3,4-dioxygenase (P34Os) (Guzik et al., 2014), and the resulting products are further metabolized into acetyl-CoA entering the TCA cycle for further oxidation and energy production. Some microorganisms (e.g., *Blastobotrys (Arxula) adeninivorans*) is capable of decarboxylating PCA by the gallic acid decarboxylase (Agdc1p) (Meier et al., 2017). The catechol-1,2-dioxygenase (Acdo1p) in *Blastobotrys raffinosifermentans* is determined to be associated with the catabolism of PCA (Meier et al., 2022). It was also found that overexpression of catechol-1,2-dioxygenase (ACDO1) in *B. raffinosifermentans* resulted in a faster conversion of catechol to *cis,cis*-muconic acid, which revealed that ACDO1 is probably associated with the PCA degradation. Furthermore, aromatic compounds such as PCA are primarily metabolized through the β-ketoadipate pathway in microorganisms. This pathway facilitates the sequential conversion of PCA into acetyl-CoA and succinyl-CoA, which serve as key intermediates for integration into the TCA cycle to drive energy production and biosynthetic processes. Representative microbial species capable of executing this metabolic route include *Rhodococcus opacus* PD630 (Navas et al., 2021) and *Aspergillus niger* (Sgro et al., 2023), both of which exhibit robust aromatic degradation capacities with potential applications in bioremediation and bio-industrial synthesis. Specially, in *Rhodococcus jostii* RHA1, a second degradation pathway for PCA was identified, which leads to the formation of hydroxyquinol (benzene-1,2,4-triol) (Spence et al., 2020). The gene cluster (ro01857-ro01860) is crucial for the degradation of PCA and it is predominantly composed of genes that encode for hydroxyquinol 1,2-dioxygenase and maleylacetate reductase. The former catalyzes the ring-cleavage of hydroxyquinol to produce maleylacetate, while maleylacetate reductase further converts maleylacetate into 3-oxoadipate. Additionally, in this cluster another two genes are hypothesized to encode a mono-oxygenase (ro01860) and a decarboxylase (ro01859).

#### 2.2.2 The metabolic pathways of EG

For EG, it could be directly metabolized into the Krebs cycle by certain microorganisms such as *Pseudomonas putida via* isocitrate (Li et al., 2019; Hosseinpour et al., 2016). In *P. putida*, this is a multi-step process, including the following critical steps: initial oxidation step, conversion to glycolic acid, generation of glyoxylic acid, and finally entry into the Krebs cycle. The alcohol dehydrogenase (ADH) plays a crucial role in the oxidation of EG, which is capable of oxidizing EG to glycolaldehyde (GA), a more reactive aldehyde intermediate. Then, GA will be catalyzed into glycolic acid catalyzed by the aldehyde dehydrogenase (ALDH). Subsequently, glycolic acid is converted to glyoxylic acid, a precursor for entry into the Krebs cycle, which is catalyzed by glycolate oxidase (GOX). Finally, the glyoxylic acid enters the Krebs cycle through the glyoxylate shunt, and this process is mainly facilitated by isocitrate lyase and malate synthase. Isocitrate lyase is capable of cleaving isocitrate (a Krebs cycle intermediate) to form glyoxylate and succinate, and the malate synthase then combines glyoxylate with acetyl-CoA to generate malate. This enzymatic process allows EG to be incorporated into the central metabolic pathway, where it would be further oxidized and utilized to generate energy in the form of ATP and reducing equivalents (e.g., NADH and FADH_2_).

*Escherichia coli* also has the capability of metabolizing EG (Shimizu and Inui, 2024). In *E. coli*, EG is firstly oxidized to GA catalyzed by propanediol oxidoreductase encoded by the *fuco* gene. Then, GA is further oxidized to glycolic acid by glycolaldehyde dehydrogenase with NAD and NADP as coenzymes. Subsequently, the glycolic acid would be metabolized *via* the glycolate pathway generating the final product glyoxylic acid, which mainly catalyzed by the glycolate oxidase. Finally, the glyoxylic acid will enter the Krebs cycle through the glyoxylate shunt similar to that found in the *P. putida*.

## 3 Strategies for the development of promising chassis cells for PET-degradation

### 3.1 Cell surface display utilized for PET-biodegradation

Cell surface display technology is a robust genetic engineering approach that facilitates the targeted anchoring of functional proteins or peptides onto microbial cell membranes through genetic engineering approaches, with applications spanning both prokaryotic and eukaryotic organisms. Its versatility stems from the ability to maintain the structural integrity and biological activity of displayed biomolecules while enabling direct interaction with extracellular environments, which makes it particularly valuable for applications ranging from whole-cell biocatalysis to environmental bioremediation and biomedical engineering. Consequently, this section highlights the strategic integration of cell surface display systems in engineering microbial catalysts for enhanced PET biodegradation, establishing this approach as a versatile platform bridging fundamental research and industrial applications.

#### 3.1.1 Application of the surface-displayed PETase in *E. coli*

**FIGURE 3:**
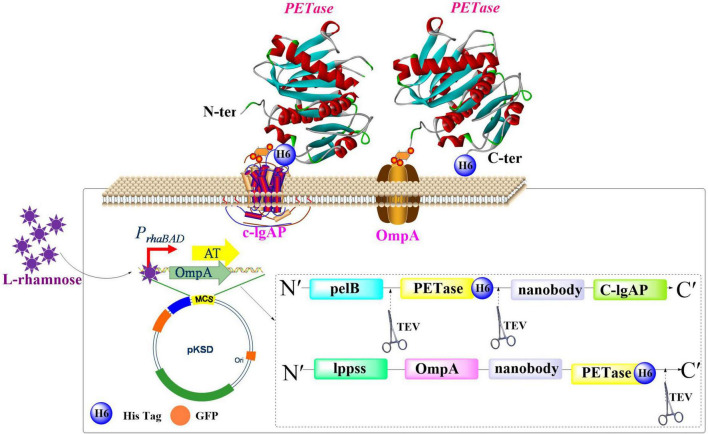
Schematic illustration of the surface display modules for heterogeneous *Is*PETase expression.

Heyde et al. (2021) proposed an efficient screening method for identifying enzymes that act on PET with surface display technology utilized in *E. coli* (Figure 3). Performance of two different membrane anchors were investigated for displaying *Is*PETase: C-IgAP from *Neisseria gonorrhoeae* and an Lpp-OmpA fusion protein from *E. coli*. Results indicated that Lpp-OmpA was more effective for displaying *Is*PETase, which displayed a PET-degrading activity comparable to 20 nM free *Is*PETase*^Austin^*. Furthermore, the fused GFP-nanobody, acting as a surrogate marker for assessing enzyme activity on the cell surface, significantly improves the efficiency of high-throughput screening strategies for identifying PET-degrading enzyme. In a separate investigation, researchers engineered *E. coli* to co-display HotPETase and a β-glucan-specific carbohydrate-binding module (*Ba*CBM) through a truncated outer membrane hybrid protein (FadL) anchoring system, which creates regenerable whole-cell biocatalysts with enhanced operational stability for PET biodegradation (Wang T. et al., 2024). This system showed enhanced PET-degradation efficiency, reusability and stability, which could produce 1.03 mM of water-soluble products, retain activity after ∼100 h at 40°C, and maintain ∼54.6% of its initial activity after nine cycles.

To achieve the purpose of efficient display of PETase, identification and design of powerful anchoring motifs is highly important. To date, several passenger proteins have been shown to be suitable for surface display of target protein. For example, PgsA, from *B. subtilis*, was shown to be capable of presenting active PETase on the cell surface of *E. coli* (Yamashita et al., 2024). YeeJ, a new inverse autotransporter in *E. coli*, has also been successfully utilized for surface display of PETase in *E. coli* UT5600 (Gercke et al., 2021). Moreover, it was found that the cell-displayed PETase in combination with externally supplied rhamnolipids displayed improved PET-degrading performance (increased by ∼16-fold) compared with the free PETase. Notably, the system demonstrated efficient degradation of highly crystalline post-consumer PET bottles (initial crystallinity: 34.4%), which results in a remarkable reduction to 8% residual crystallinity within 3 days under mild conditions (25°C). The whole-cell biocatalyst system featuring surface co-display of high-affinity adhesive proteins (cp52k from the stalked barnacle *Pollicipes pollicipes* and mfp-3) and Fast-PETase has been successfully constructed in *E. coli* to enhance PET biodegradation efficiency (Hu and Chen, 2023). The ice nucleation protein (INP), a structurally characterized outer membrane protein derived from *Pseudomonas syringae*, has also been extensively employed as an anchoring scaffold to enable effective surface display of fused functional proteins (Ramezani Khorsand et al., 2024). This application leverages its modular architecture comprising N-terminal membrane-anchoring domains, repetitive central β-helical motifs for water interaction, and C-terminal aggregation interfaces.

**FIGURE 4:**
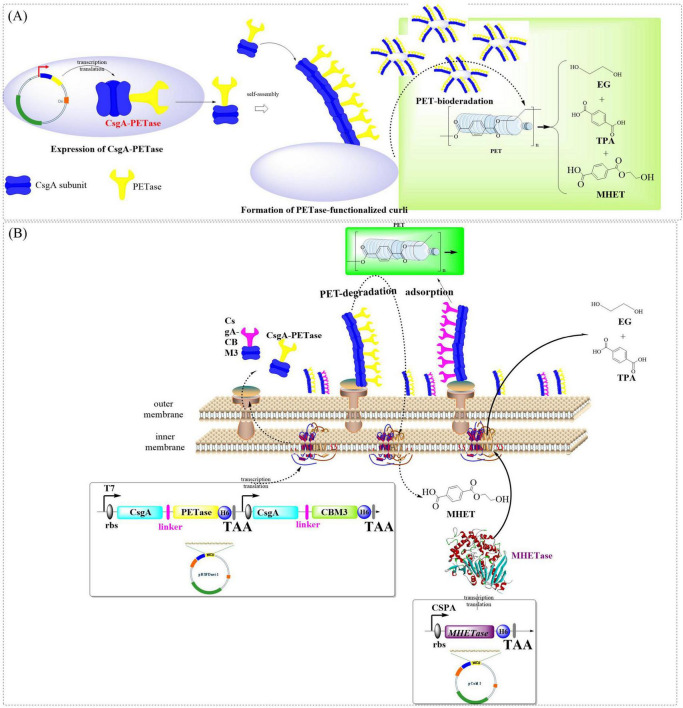
Schematic illustration of the engineered curli of *E. coli* by fusing CsgA with *IsP*ETase for PET degradation. (**A**: Construction of the whole-cell biocatalyst BINDPETase; **B**: strategic intracellular co-expression of PETase coupled with surface-displayed CBM3).

Curli, composed of self-assembling CsgA monomers, is capable of forming β-sheeted nanofibers covering the bacteria, which might be also utilized to facilitate the secretion and display of catalytic enzymes (Sleutel et al., 2023; Zhang X. et al., 2023). Recently, a proposal has been put forward to engineer the curli of *E. coli* by fusing CsgA with *Is*PETase for PET degradation (Zhu et al., 2022). The fused CsgA-PETase was capable of forming functional PETase-coated curli nanofibers on the surface of *E. coli* PHL628, which resulted in the formation of whole-cell biocatalyst (BIND-PETase, Figure 4A). BIND-PETase exhibited an impressive activity of 966 ± 29 U/g and demonstrated remarkable stability even after 30 days at both 4°C and room temperature. However, it is worth noting that its activity dropped to 55% after only three cycles. BIND-PETase could effectively degrade PET microplastics in wastewater and depolymerize post-consumer PET releasing TPA and MHET (total of 4.3 mM after 15 days, and 3.3 mM after 7 days, respectively). Especially, the degrading performance could be further improved by 22.1% with addition of 0.02% STS. Similarly, fusing CsgA and PETases to *E. coli* Nissle 1917 (EcN) resulted in a 10-fold increase in PET-degrading activity (Wang C. et al., 2024) compared to that from Zhu et al. (2022). Among the obtained whole-cell catalysts, EC-FastPETase shows the highest activity in PET film degradation [3.0 ± 0.073 mg/(d × cm^2^)] with a total product of 11.4 ± 0.3 nM. Especially, co-display of CBM3 and EC-FastPETase significantly promoted biofilm formation and cell adsorption resulting a 35% increase in degrading activity (3.4 ± 0.148 mg/(d × cm^2^), yield 1.5 ± 0.1 mM). This reaction system was also capable of effectively degrading PET film and microplastics, releasing 12.7 ± 0.2 mM TPA and 1.65 ± 0.05 mM MHET. In addition, it could catalyze the transformation of 10 and 100 μm PET microplastics into TPA (11.14 ± 0.12 and 8.38 ± 0.1 mM), with degradation efficiencies of 21.40% and 16.09%, respectively. The improved catalytic efficiency could be partially attributed to the enhanced secretory capacity of extracellular proteins. More significantly, the strategic intracellular co-expression of MHETase coupled with surface-displayed CBM3 synergistically enhanced both substrate accessibility and metabolic processing of PET polymers (Figure 4B). This integrated enzymatic configuration demonstrated remarkable catalytic enhancement, achieving a 10- to 20-fold greater degradation efficiency for BHET compared to the conventional free enzyme systems.

#### 3.1.2 Application of the surface-displayed PETase in yeast cells

**FIGURE 5:**
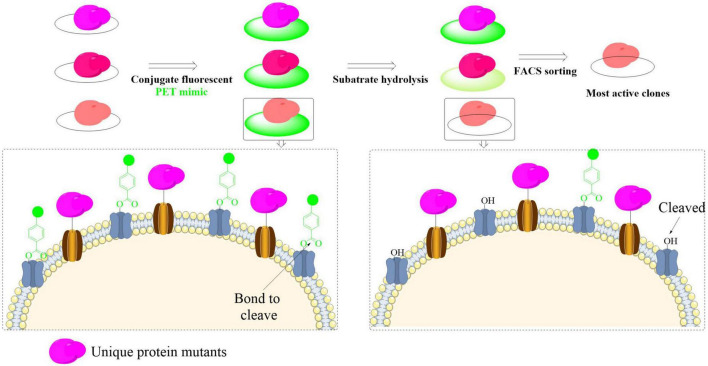
Schematic illustration of the high-throughput yeast surface display platform for efficient identification and evaluation of PET-degrading enzymes.

A state-of-the-art yeast surface display system with ultra-high-throughput capabilities has recently been engineered to streamline the discovery and functional characterization of PET-hydrolyzing enzymes (Cribari et al., 2023). The system demonstrates exceptional efficiency in enzyme evaluation through simultaneous display of multiple enzyme variants on yeast cell surfaces, allowing quantitative assessment of binding interactions and catalytic performance without requiring protein purification. It involves three critical steps: design of a PET-mimicking probe for yeast surface display, utilization of fluorescent streptavidin to label unhydrolyzed probes, and use of the fluorescence activated cell sorting (FACS) to identify and obtain high-activity catalysts (Figure 5). Using this method, a library of 2.7 million yeast clones was screened, especially it was found that mutations at H218 could enhance PET-degrading activity. This was further confirmed by the mutant H218Y with significantly increased PET degradation (by approximately twofold). The observed improvement arose *via* two synergistic effects: (1) direct non-covalent interactions of Y218 with the PET substrate, and (2) the subsequent enhancement in enzyme-substrate binding affinity. However, while surface-displayed PETase demonstrates remarkable catalytic turnover enhancement, studies have revealed critical limitations in practical implementation of whole-cell catalysts that exhibit optimal functionality only at extremely low concentrations (typically < 0.1 mg/L) (Chen et al., 2020). This concentration-dependent performance constraint, particularly evident in heterogeneous catalytic systems, poses significant challenges for scaling up PET biodegradation processes in real-world environments where high catalyst loading and operational stability are essential. Overall, this strategy could efficiently address critical limitations in traditional PETase screening methods that often rely on surrogate soluble substrates. Moreover, it also enables precise control over enzyme-substrate ratios through cell-surface engineering strategies.

It must be noted that the N-linked glycosylation might pose negative effects on the *P. pastoris*-expressed recombinant protein (Han et al., 2020; Huang et al., 2013). It was estimated that N-glycosylations in eukaryotic expression systems could interfere with the bio-activity of prokaryotic genes. This is extremely true for the recombinant *Is*PETase and *Bur*PL expressed in *P. pastoris*, although the decreased catalytic activities could be further restored by de-glycosylation treatment or mutagenesis (Chen et al., 2021). Similarly, it was also found that N-glycosylation at N181 in *Ct*PL-DM, a PET-degrading enzyme from *Caldimonas taiwanensis*, would significantly reduce its catalytic activity when expressed in *P. pastoris* (Li et al., 2023b). This might result in a putative N-glycosylation site N181, which restrained the conformational change of the substrate-binding Trp. However, the catalytic activity could also be restored by removing the N-glycosylation such as through the N181A mutation. Further de-glycosylation at additional sites (N181A, N220A, and N261A) show little effect on the performance of N181A, which further underscores the importance of the residue N181. Based on these findings, performance of N181A was further enhanced *via* molecular engineering. The resulting CC mutant (*Ct*PL-DM^*R*230*C/S*284*C*^) and the *Ct*PL-DM^*N*181*A*/*F*235*L*^ mutant showed over 60% and ∼20% increased PET-degrading activity at 60°C, respectively. Notably, CC variant demonstrated higher catalytic activity at elevated temperatures, with a 2.5-fold increase at 70°C compared to 60°C. After 5 days, the *Ct*PL-DM^*N*181*A/F*235*L*^-mediated degradation of amorphous GfPET film and reinforced PET resulted in a total of monomeric products of ∼5 mM and 0.8 mM at 50°C, respectively.

The structural incompatibility between yeast glycosylation machinery and bacterial enzymes poses functional challenges in heterologous expression systems. Specifically, yeast platforms (e.g., *Yarrowia lipolytica*) exhibit yeast-specific N-glycosylation patterns that might disrupt the folding and catalytic activity of bacterial-derived PET hydrolases like *Is*PETase. The industrial-scale implementation of microbial PET degradation systems also encounters significant technical challenges, primarily stemming from ineffective maintenance of extracellular pH levels that severely impair enzymatic functionality and catalytic stability. This issue could be compounded by undesirable metabolic flux diversion toward ethanol accumulation and non-productive byproduct biosynthesis pathways. To address these challenges, codon optimization, utilization of glycosylation-reduced strains, and fed-batch fermentation strategies optimized for PETase production would be prioritized.

#### 3.1.3 Application of the co-display systems in engineered cells

**FIGURE 6:**
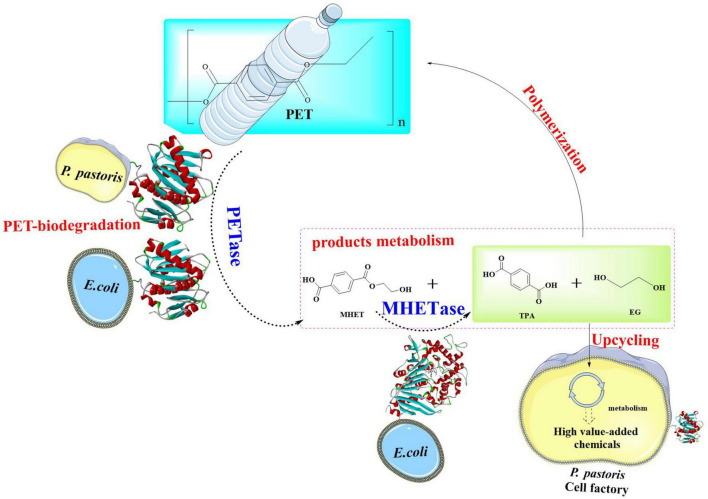
Graphic representation of surface co-display platforms with engineered PETase and MHETase on both *E. coli* and *P. putida*.

Recently, a novel strategy was proposed involving the surface co-display of engineered PETase variants and MHETase on both *E. coli* and *P. putida* platforms (Figure 6; Xue et al., 2024). Fluorescence quantification identified YfaL as the most efficient autotransporter among the tested variants (YfaL, YeeJ, Aom, and EstP), which exhibits a 4.2-fold higher signal intensity compared with that of YeeJ. It is further confirmed that the engineered strains co-displaying DuraPETase and MHETase exhibited enhanced biocatalytic activity against both BHET and PET substrates, especially the strain PD5 achieves exceptional BHET degradation rates over 1 mM/h. Especially, critical evaluation of catalytic kinetics revealed DuraPETase as the rate-limiting enzyme governing PET depolymerization efficiency in the dual-enzyme cascade system. Therefore, to enhance the surface display of DuraPETase, two distinct strategies were implemented. The initial experimental setup involved two genetically engineered bacterial strains: UTD1 (lacking the outer membrane protease OmpT, a key virulence factor implicated in host cell adhesion and antimicrobial peptide degradation) and ELD1 (with an inactivated *lpp* gene to promote enhanced outer membrane vesicle production, a modification associated with compromised envelope integrity). Second, molecular chaperones were co-expressed under inducible promoters in strains ECD1 and ELCD1. These strategies significantly improved the PET-degrading activities, especially co-expression of chaperones in ECD1 resulted in the highest catalytic activity, achieving 1.82 mM/h on BHET and 0.302 mM/day on PET powder. However, combining all strategies in strain ELCD1 did not yield higher activity as expected, which highlights the complexity of optimizing surface display efficiencies. Based on these, engineering of the YeeJ translocator domain resulted in mutant E9D1 (YeeJ^*I*42*F/K*105*N/K*106*N/D*116*G/S*294*G/N*78*S/E*293*V*^) and E33D1 (YeeJ^*I*42*F*/*K*105*N/K*106*N/D*116*G/S*294*G/N*197*K/D*358*C*^), which displayed significantly higher PET degradation rates. It is suggested that reduced chain length and interaction forces were capable of facilitating secretion efficiency. For example, disruption of the salt bridge (N78S/E293V) in E9D1 increased β-barrel conformational entropy to enhance substrate threading, while charge-swap mutations (N197K/D358C) in E33D1 electrostatically dilated the translocation pore by 2.1 Å. These synergistically optimized the secretory flux. Especially, strain EC9F exhibited a remarkable PET degradation rate of 3.85 mM/day, with a 51-fold increase over the initial strain ED1. Under the optimal conditions, this dual-enzyme cascade system achieved the highest reported PET degradation rate of 4.95 mM/day.

**FIGURE 7:**
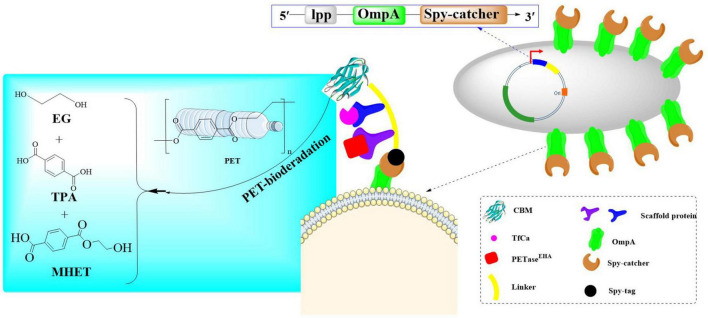
Diagrammatic representation of a robust whole-cell biocatalyst constructed with the PT-EC biocatalytic complexes displayed on the *E. coli* surface *via* coordinated Lpp-OmpA membrane fusion and SpyCatcher/SpyTag covalent immobilization.

Recently, a PT-EC enzymatic consortium was engineered in *E. coli* BL21(DE3) through a modular assembly strategy incorporating the dual-mutant LCC^*F*242*A/D*238*C*^ variant, TfCa carboxylesterase, and crystalline cellulose-binding module CBM3, which exhibits a 2.7-fold enhancement in polyethylene terephthalate (PET) depolymerization efficiency compared to conventional enzymatic systems (Aer et al., 2024a). The system strategically employs a biomimetic cellulosome-inspired scaffolding architecture, which combines a chimeric cohesin-TB hybrid scaffold with heterologous dockerin domains to establish a spatially organized multi-enzyme assembly. This design is able to enhance the proximity-driven catalytic synergism through optimized spatial arrangement of enzymatic components. In this way, a <2 nm inter-enzyme spacing within the scaffolded complex could be developed, and a 3.8-fold increase in PET-to-TPA conversion efficiency could be achieved. Finally, the PT-EC biocatalytic complexes (Figure 7) were precisely anchored on the *E. coli* surface *via* coordinated Lpp-OmpA membrane fusion and SpyCatcher/SpyTag covalent immobilization. The developed whole-cell biocatalyst exhibited remarkable thermal stability and solvent adaptability, maintaining over 80% of its original catalytic activity even after prolonged incubation at 50°C, while demonstrating exceptional tolerance to a wide range of organic solvents including polar and non-polar systems. At 40°C and pH 7.4, PT-EC^*EHA*^ achieved 16.5 ± 1.2 mM TPA accumulation within 48 h, which significantly surpassed that of PETaseEHA (1.0 ± 0.3 mM). Furthermore, the surface-displayed PT-EC^*EHA*^ further exhibited a PET film mass loss rate of 2.3 mg/cm^2^/day, a 20-fold improvement over the free enzyme system. In a 7-day evaluation at 40°C, PT-EC^*EHA*^ demonstrated exceptional catalytic efficiency with a 4.3 mM increase in product release. While PT-ECZPE exhibited significantly lower degradation activity on PET films (≤5% surface erosion), it demonstrated markedly enhanced performance with amorphous PET powder substrates. Notably, the hydrolytic products (TPA/EG) showed sustained linear release kinetics during 7-day incubation, reaching cumulative yields of 11.56 mM (*R*^2^ = 0.98). This dichotomy directly demonstrates that substrate crystalline structure and enzyme accessibility are critical limiting factors in enzymatic PET depolymerization processes. Finally, through combinatorial mutagenesis screening, a thermostable variant TfCa^*I*69*W/L*281*Y*^ was identified, which demonstrated a 2.5-fold enhancement in the accumulation of products (TPA/EG). Notably, it could maintain >90% residual activity after 8 h at 65°C (*t*_1/2_ = 15.3 h) with specific activity reaching 38.7 U/mg. This clearly revealed MHET intermediate accumulation could be effectively alleviated during extended degradation processes.

*Escherichia coli* platforms (e.g., with Lpp-OmpA-anchored *Is*PETase) excel in rapid engineering, scalability, and compatibility with synthetic biology tools. However, proteolytic degradation (e.g., OmpT-mediated cleavage) and insufficient glycosylation limit their long-term stability. Yeast systems (e.g., *P. pastoris* displayed PETase) bypass protease issues and enable eukaryotic post-translational modifications. However, unintended N-glycosylation (e.g., at residue N181 in CtPL-DM) might result in the reduced catalytic activity unless mitigated *via* mutagenesis (e.g., N181A). In conclusion, *E. coli* remains preferable for scalable whole-cell biocatalyst production, while yeast systems are better suited for eukaryotic enzyme optimization.

### 3.2 Optimizing the metabolic pathways of the host for PET biodegradation

#### 3.2.1 Metabolic engineering of *P. putida*

Recent breakthroughs in synthetic biology have enabled the development of *P. putida* strains with dual catalytic capabilities: (1) efficient assimilation of PET monomers *via* customized metabolic pathways, and (2) robust extracellular secretion of functional PET hydrolases through engineered secretion systems (Brandenberg et al., 2022). This integrated approach leverages modular genetic circuits for monomer metabolism while employing Type II secretion machinery to achieve coordinated enzyme production and extracellular release. It started with chromosomal integration of the *tphII* operon from *Comamonas* sp. E6 into *P. putida* KT2440 driven by a constitutive promoter. Subsequent optimization of TA assimilation was achieved through adaptive laboratory evolution (ALE) coupled with targeted mutagenesis of the MhpT transporter system. To address transcriptional repression mediated by the GclR regulator on glycolate (Gcl) and glyoxylate (GlxR) metabolism, parallel ALE campaigns were also conducted. *P. putida* TA7-EG with a nonsense mutation in *gclR* (PP_4283) resulted in truncated GclR protein and de-repressed C2 metabolism. *P. putida* TA7-BD acquiring a missense mutation in PP_2046 (putative transcriptional regulator) shows significantly enhanced 1,2-butanediol (BD) utilization efficiency. Building upon this foundation, a modular surface display system was engineered using different membrane anchors (EhaA secretion system, InaV ice nucleation protein, and OprF porin) for extracellular PET hydrolase presentation. In addition, the chimeric architecture also contained: (1) OprF signal peptide for periplasmic translocation; (2) catalytic domains (HiC, LCC, or *Is*PETase mutants); (3) flexible glycine-serine linker (G4SGGS(G4S)3); and (4) C-terminal transmembrane anchor. These are all under the regulation of rhamnose-inducible P_*rhaB*_ promoter. Results revealed *P. putida* TA7-EG transformants exhibited 1.3–2.5-fold enhanced esterolytic activity, and the OprF-HiC chimera demonstrated peak performance as 2.68 ± 0.15 U/ml. Notably, rhamnose-induced expression triggered significant growth defects (30%–45% biomass reduction), particularly in LCC-expressing strains, which might highlight the enzyme-specific cytotoxicity. Moreover, to achieve temperature-responsive transcriptional regulation of heterologous expression, two thermoregulated systems were engineered: (1) the λ phage-derived cI857/PL system employing a thermosensitive repressor, and (2) a synthetic P_*IbpA*_ variant incorporating *Pseudomonas aeruginosa* IbpA-5′UTR modifications. It was shown that the engineered P_*IbpA*_ enabled robust PET hydrolase production (0.92 ± 0.08 mg/L extracellular protein) with minimal growth compromise (12% biomass reduction). The pLo-P_*IbpA*_-EhaA-LCC recombinant system exhibited temperature-responsive activation kinetics, achieving a 10^4^-fold increase in esterolytic activity (212.4 ± 8.7 U/mg) at 30°C, which is a 37% improvement over λ P_*L*_-regulated counterparts. Notably, this performance was optimized through a controlled temperature gradient protocol (25–30°C for 6 h), which effectively mitigated proteotoxic stress-induced biomass loss from 45% to 12% while preserving extracellular PET hydrolase activity at 212.4 ± 8.7 U/mg. This temperature-modulated expression strategy aligns with recent advancements in enzyme thermal stability engineering, where gradual thermal adaptation has been shown to enhance both catalytic efficiency and cellular viability in heterologous expression systems. The optimized method demonstrated remarkable efficacy in protein aggregation control, achieving a 2.3-fold reduction in misfolded protein accumulation. Moreover, it effectively maintained cellular viability at over 85%, while simultaneously addressing the growth defects characteristic of LCC-expressing strains through direct intervention in aggregation-related pathways. Based on these, chromosomal integration of PIbpA-PET hydrolase expression cassettes in the engineered *P. putida* strains was achieved. While this genomic integration strategy effectively balanced hydrolase expression with cellular viability, it resulted in a concomitant reduction in overall enzyme production levels. Notably, *P. putida* KT2440 carrying the P_*IbpA*_-bglx-HiC construct achieved a 25% mass reduction in PBAT-starch-PLA copolymer films within 6 days. When extended to a 4-week incubation period, the engineered strain *P. putida* TA7-BD expressing the same enzyme system mediated approximately 40% depolymerization efficiency for the copolymer substrate. Although the PET upcycling cell factory constructed in this study demonstrated limited autotrophic efficiency, it uncovered critical challenges in engineering robust microbial chassis systems for industrial-scale applications. The study revealed three key engineering challenges in heterologous hydrolase expression systems, including (1) growth impairment caused by metabolic burden, (2) host protease-dependent enzyme degradation, and (3) suboptimal transmembrane transport of polymeric substrate degradation intermediates. The enduring challenges inherent in microbial metabolic networks highlight fundamental limitations demanding systematic investigation, particularly focusing on: (1) carbon flux maldistribution during the integration of heterologous pathways, (2) insufficient regeneration capacity of energy currencies (ATP/NADPH) under suboptimal metabolic states, and (3) redox imbalance triggered by exogenous enzymatic systems.

**FIGURE 8:**
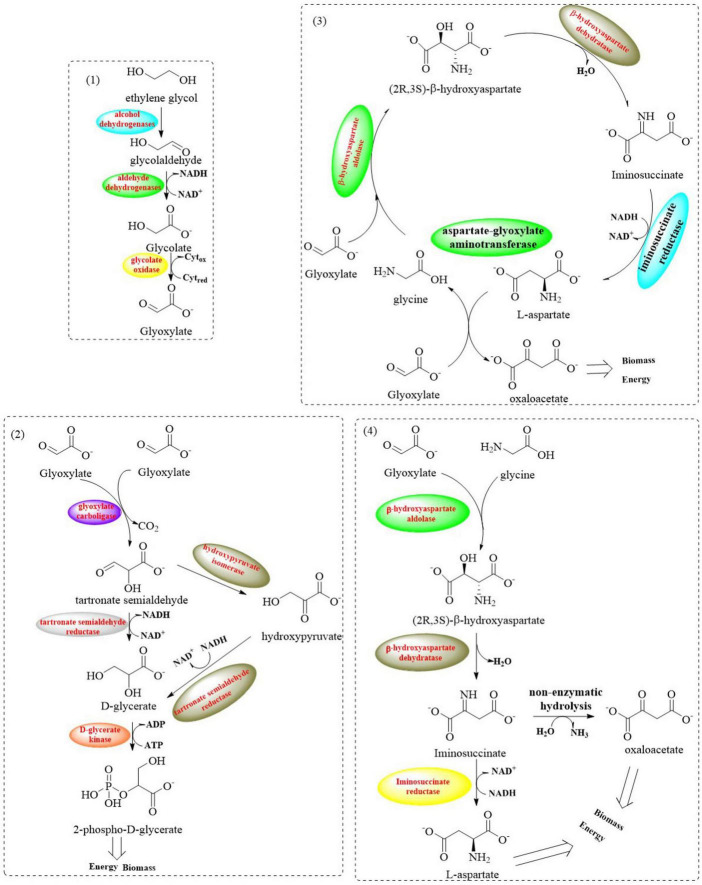
Graphical representation of metabolic pathways involved in EG assimilation (1: oxidative reactions from EG to glyoxylate; 2: the glycerate pathway: it converts two glyoxylate molecules into two – phosphoglycerate and CO_2_; 3: the BHAC: a cyclic pathway where four enzymes transform two glyoxylate molecules into one oxaloacetate molecule; 4: the BHA shunt: it convertes glyoxylate and glycine into aspartate).

Extensive biochemical studies have established that most microbial systems employ the canonical glycerate pathway for EG assimilation (Mückschel et al., 2012; Franden et al., 2018; Werner et al., 2021). This oxidative metabolic route involves sequential enzymatic conversions through intermediates like glycolaldehyde (GA) and glyoxylate, ultimately yielding CO_2_ as the terminal oxidation product with suboptimal carbon efficiency. However, this process necessitates supplementary reducing equivalents (NADH/FADH2) and ATP, which poses critical industrial bottlenecks through three interconnected mechanisms: (1) growth arrest triggered by redox cofactor depletion, (2) pathway flux oscillations induced by energy deficits, and (3) cumulative metabolic stress from auxiliary enzyme overexpression. Recent advances in synthetic metabolism have revealed the β-hydroxyaspartate cycle (BHAC) as a biochemically streamlined route that bypasses decarboxylation steps while preserving carbon fidelity (Figure 8; Schada von Borzyskowski et al., 2019; Roell et al., 2021). It involves a coordinated system comprising four key enzymes encoded by the *Bhc* gene cluster: the PLP-dependent aminotransferase (BhcA), serine/threonine dehydratase (BhcB), β-hydroxyaspartate aldolase (BhcC), ornithine cyclodeaminase (BhcD), and along with an IclR-family transcriptional regulator. This multi-enzyme complex catalyzes the efficient conversion of two glyoxylate molecules into oxaloacetate through an energy-conserving mechanism that requires only one NADH equivalent while maintaining complete carbon retention (without CO_2_ release) (Schada von Borzyskowski et al., 2019).

**FIGURE 9:**
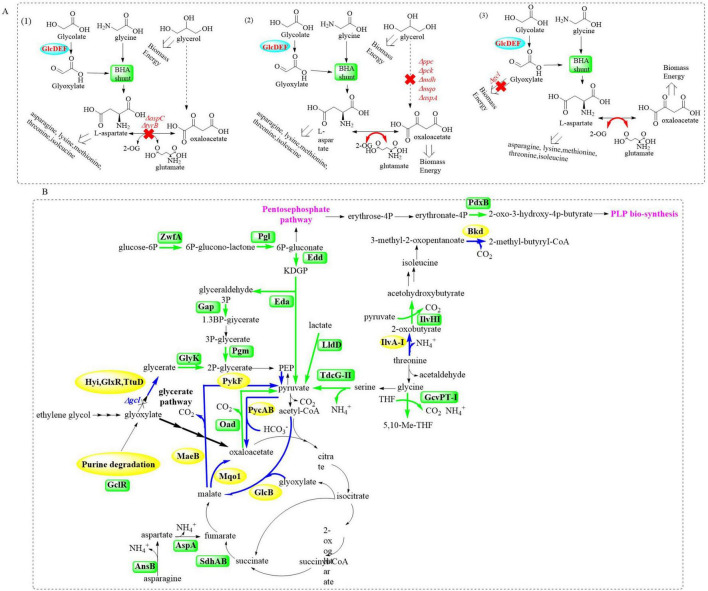
Graphical representation of the strategic engineering for glycerate metabolism by introducing the BHAC pathway in *E. coli* **(A)** and *P. putida* KT2440 **(B)**.

Based on these mechanistic insights, the potential of implementing the BHAC pathway in *P. putida* KT2440 was investigated through strategic engineering of glycerate metabolism (Schada von Borzyskowski et al., 2023). Core enzymes (BhcC/BhcB/BhcD) were first tested in *E. coli* chassis strains with incremental metabolic demands (Figure 9A). Glycolate was selected over toxic glyoxylate, leveraging endogenous conversion capacity. In aspartate-auxotrophic ΔaspC/ΔtyrB *E. coli* grown on glycerol/glycolate/glycine, the full BHA shunt enabled growth (18.2 ± 0.4 h doubling), whereas pathway-deficient controls failed. Tyrosine limitation was identified as the bottleneck, alleviated by 1 mM supplementation (13.0 ± 0.4 h doubling), which indicates precursor flux redirection might compensate the auxotrophy.

Based on the initial testing in *E. coli*, structure-guided engineering of *P. putida* KT2440 was employed to replace native glycerate metabolism with the BHAC pathway aiming to enhance PET degradation capabilities while maintaining the metabolic robustness. Upon integration of the complete BHAC pathway into *P. putida* KT2440 Δ*gcl* (Figure 9B), the engineered strain *P. putida* Δ*gcl* + BHAC demonstrated immediate growth initiation on EG-containing medium, bypassing the adaptive lag phase required by its counterpart. Enzymatic assays revealed activities of the heterologous BHAC pathway components at ∼0.5 and 10 U/mg, which highlights the distinct catalytic efficiencies between key enzymes. Furthermore, the BHAC-equipped strain exhibited a 5%–10% increase in growth rate compared to *P. putida* KT2440 E6.1, a derivative previously optimized via ALE to metabolize EG through the native glycerate pathway. This enhancement underscores the metabolic superiority of the synthetic BHAC route over the naturally evolved glycerate-dependent system in terms of carbon utilization efficiency and kinetic performance.

Proteomic profiling of the strain *P. putida* Δgcl + BHAC uncovered a systemic metabolic rewiring, which is characterized by the following points. (1) The downregulation of glycolytic/gluconeogenic nodes (PycAB, Mqo1, and MaeB) coupled with upregulation of oxaloacetate decarboxylase (Oad), which establish oxaloacetate as the predominant entry point for TCA cycle influx. (2) Diverting carbon flux from glyoxylate shunt through decreased malate synthase (GlcB) activity and elevated succinate dehydrogenase (SdhAB) levels. This reveals preferential routing of acetyl-CoA through canonical TCA oxidation rather than bypass mechanisms. (3) Aspartate-to-TCA rechanneling *via* upregulated aspartate transaminases, which enables nitrogen-efficient α-ketoglutarate synthesis. (4) Activation of glycine salvage pathways is achieved through enhanced glycine cleavage system components, which is capable of facilitating serine/pyruvate interconversion to maintain redox balance. (5) Cofactor provisioning *via* PdxB-mediated pyridoxal 5′-phosphate (PLP) biosynthesis is associated with creating a self-reinforcing loop to sustain BHAC enzyme activity and biomass accretion. This multipronged adaptation highlights the metabolic prioritization of BHAC-mediated EG assimilation over native routes, which would achieve energy and redox balance through streamlined carbon funneling into the TCA cycle.

Finally, to further optimize chassis performance, ALE was applied to *P. putida* KT2440 Δgcl + BHAC. The evolved strain demonstrated a 35% increase in specific growth rate (μ = 0.42 ± 0.03 h^–1^) and 20% higher maximum biomass density (OD_600_ 8.7 ± 0.2) under 20 mM EG. Genomic analysis identified subtle mutations in the *bhcABCD* expression module and key genomic adaptations. It mainly involve: (1) regulatory adjustments caused by a LysR-family transcriptional regulator (*lldR*) mutation and altered expression of lactate utilization and glycolate metabolism regulators (*regB*); (2) redox rebalancing resulting from the upregulated *lldD* (lactate dehydrogenase) and *dld2* (D-lactate dehydrogenase), driven by the *lldR* mutation capable of modulating pyruvate-lactate interplay; (3) systemic optimization including the enhanced RegAB (redox sensors) and CioA (cytochrome oxidase) expression, the remodeling of electron transport chain and the fine-tuned central carbon/amino acid metabolism. These multi-tiered adaptations synergistically enhanced redox homeostasis and substrate channeling into the TCA cycle, maximizing EG assimilation. This work establishes a chassis with engineered metabolic robustness for PET-derived EG upcycling, bridging biodegradation and sustainable chemical synthesis.

Recent scientific advances have demonstrated an innovative closed-loop strategy for plastic waste valorization through biocatalytic cascade reactions (Tiso et al., 2021). The post-consumer PET was successfully converted into two structurally distinct biodegradable polymers: microbial-derived polyhydroxyalkanoates (PHAs) and a novel bio-based poly(amide urethane) (bio-PU) synthesized through chemoenzymatic modification. The integrated biorecycling process begins with enzymatic depolymerization of post-consumer PET films using an engineered LCC variant in a thermostated stirred-tank reactor (70°C). Kinetic analysis showed peak TPA and EG production rates of 4.1 ± 0.3 and 2.1 ± 0.2 mM⋅h^–1^, respectively, within the first 24 h. Gravimetric and HPLC analyses confirmed 47.2% ± 2.1% depolymerization efficiency with near-complete surface erosion (92%–95%), while bulk crystallinity remained stable (X_*c*_ > 0.45) over 120 h. However, an 18.7% efficiency decline was observed between 24 and 120 h, which was attributed to synergistic factors: (1) arrhenius-driven thermal deactivation of LCC (E_*a*_ = 58.2 kJ⋅mol^–1^) and (2) TPA-induced acidification (ΔpH 1.8) causing non-competitive inhibition, as evidenced by 3.2-fold *K*_*m*_ increase and 68% *V*_*max*_ reduction. These results enabled development of two distinct metabolic pathways in *Pseudomonas* species for bioplastic monomer biosynthesis. Firstly, it was confirmed that that PET hydrolyzate showed no growth inhibition on *P. putida* KT2440 and *Pseudomonas umsongensis* GO16, moreover the latter showed enhanced growth possibly caused by the capability of utilizing TPA as an additional carbon source. The metabolic engineering of *P. umsongensis* GO16 for enhanced EG utilization was accomplished through carbon source-directed ALE under EG-limiting conditions. The optimized strain KS3 exhibited remarkable performance, achieving complete EG assimilation (1.7 ± 0.2 g/L) within 9 h while demonstrating a maximum specific growth rate (μ_*max*_) of 0.32 h^–1^. Upscaling to 5-L bioreactors revealed distinct substrate utilization patterns, achieving complete TPA depletion within 23 h (q_*TPA*_ = 0.45 g/gCDW/h) compared to extended EG consumption (q_*EG*_ = 0.13 g/gCDW/h). The process yielded 2.3 g/L biomass, with nitrogen limitation inducing PHA accumulation reaching 0.15 g/L (6.5% cell dry weight). The final yield reached 0.21 g CDW/g substrate, with the C8–C12 PHA profile closely matching that of the TPA-fed controls, which thereby confirmed the metabolic fidelity. Furthermore, it was elucidated that EG might be primarily routed to *de novo* fatty acid synthesis due to its high redox potential, while TPA required complementary pentose phosphate/TCA pathways. Then, the genetically engineered *P. umsongensis* GO16 KS3 was used to produce HAAs, reaching a concentration of 35 mg/L. The strain preferentially synthesized HAAs from TPA and utilized EG for growth, with a production rate of 5 mg/L/h and a yield of 0.01 g_*HAA*_/g_*TPA*_. The obtained HAAs were confirmed to be a mixture of four congeners with hydroxydecanoate as the main hydroxy fatty acid. This process is believed to not only diverts PET from waste streams but also produces a bioplastic with diverse applications, showcasing an effective circular economy approach.

In a similar study, PET oligomers derived from chemical recycling were fully converted into monomers through bio-depolymerization, which were then utilized for the production of PHAs by a co-culture of two genetically modified microbes (Liu P. et al., 2023). Firstly, *E. coli* BL21 (DE3)-LCC^*ICCG*^ was used as the chassis cell to express the PET hydrolase LCC^*ICCG*^ capable of degrading the PET oligomers into monomers, and the continuous metabolism of these produced oligomers. It was shown that BL21(DE3)-LCC^*ICCG*^ is able to depolymerize 76.9% of the PET oligomers into 0.61 g/L MHET, 5.48 g/L TPA, and 2.87 g/L EG after 72 h of cultivation at 30°C. Especially, within 24 h, BHET could be fully degraded.

**FIGURE 10:**
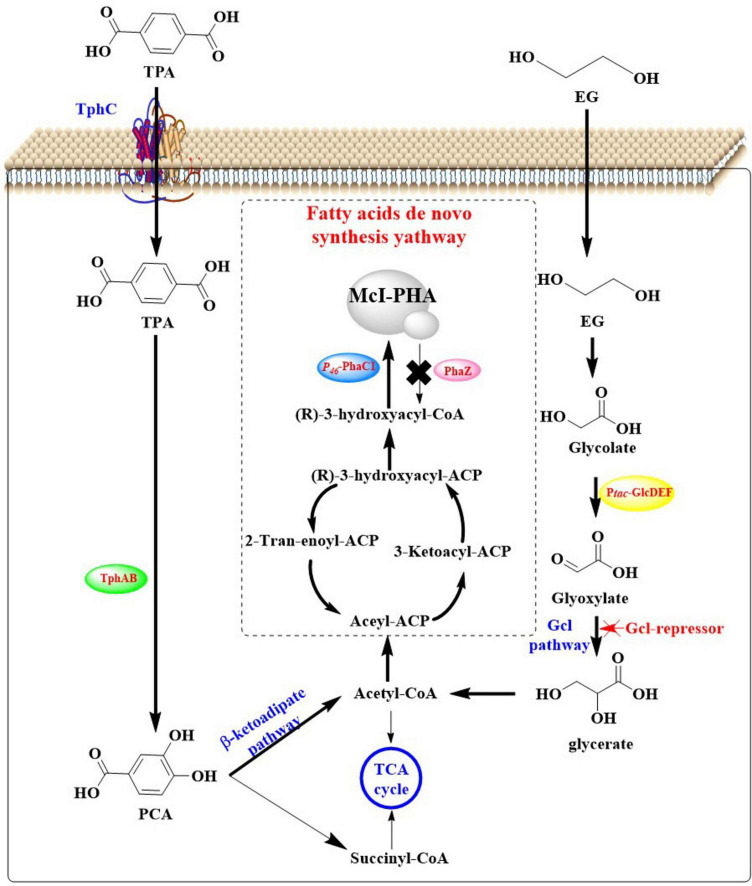
Graphical representation of the co-culture of genetically modified *E. coli* BL21 (DE3)-LCC^ICCG^ and *P. putida* KT2440-Δ*RDt-*Δ*ZP46C* for the biodegradation and up-cycling of PET.

Given that MHET was determined to be an inhibitor for LCC in the PET degradation (Barth et al., 2016), the helper enzyme *Is*MHETase was therefore expressed in *P. putida* KT2440 to accelerate the hydrolysis of MHET into TPA and EG. Moreover, the native EG metabolic pathways of *P. putida* KT2440 were improved by the deletion of *gclR* gene, the overexpression of glycolate oxidase gene and the insertion of *tph* gene cluster. The resulting stain *P. putida* KT2440-ΔRDt (Figure 10) could grow rapidly with TPA and EG as the only carbon sources, and the OD_600_ reached 14.77 after 74 h. By this strain, 16.67 g/L TPA could be completely metabolized within 74 h with a catabolic rate of 0.22 g/(L⋅h). About 1.37 g/L PHA could be obtained from 16.67 g/L TPA and 6.08 g/L EG, furthermore the yield could be increased to 1.53 g/L after deletion of the endogenous PHA depolymerase gene *phaZ* (forming *P. putida* KT2440-ΔRDt-ΔZ). Moreover, overexpression of PHA synthase (*phaC*1) under the control of P_46_ revealed that the obtained *P. putida* KT2440-ΔRDt-ΔZP46C is able to catalyzed the formation of 1.90 g/L PHA. Then, the two engineered host were applied for the biosynthesis of PHA from PET oligomers in one pot, and a low level of MHET (0.28 g/L) was detected at 12 h during the co-cultivation process. The yield of PHA was 1.10 g/L with a conversion rate of 0.11 g_*PHA*_/g_*oligomer*_, and the products were mainly consisted of 3-hydroxydecanoate (60.88%) and 3-hydrox-yoctanoate (23.67%). This conversion rate might be the currently highest level for the biosynthesis of utilizing PET or PET monomers as substrate.

#### 3.2.2 The application of synthetic biology in the construction of chassis cells for PET up-recycling

**FIGURE 11:**
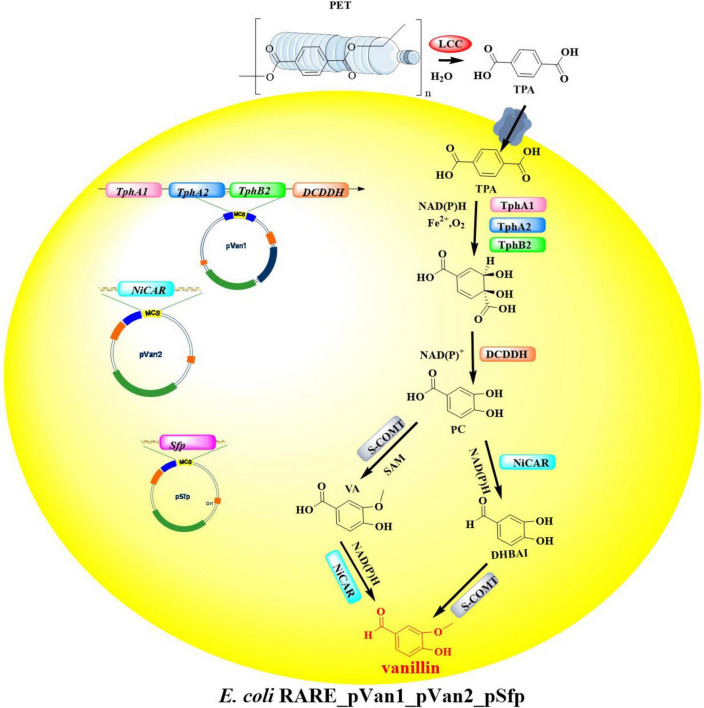
Diagrammatic illustration of the engineered metabolic pathway for the bioconversion process of TPA.

Integrated with emerging PET degradation and recycling technologies, the strategic repurposing of recovered monomers for synthesizing new PET materials or high-value derivatives presents a promising sustainable solution. To advance circular economy objectives in plastic waste management, a biosynthetic platform in engineered *E. coli* MG1655 RARE was developed that enables direct biological conversion of PET-derived TPA into vanillin-a commercially significant flavor compound and chemical precursor (Sadler and Wallace, 2021). In this engineered metabolic pathway, four key enzymes are essential for the bioconversion process (Figure 11): terephthalate 1,2-dioxygenase (TPADO), dihydroxy-3,5-cyclohexadiene-1,4-dicarboxylic acid dehydrogenase (DCDDH), carboxylic acid reductase (CAR), and catechol O-methyltransferase (COMT). For the biotransformation of TPA to vanillin, recombinant *E. coli* strains were engineered to co-express three essential plasmids: pVan1 (encoding TPADO), pVan2 (harboring the *Nocardia iowensis* CAR gene, NiCAR), and pSfp (containing the phosphopantetheinyl transferase gene required for CAR activation). Results showed that with added TPA (5 mM), a much lower yield of vanillin (5 μM, <1% conversion) was achieved. Notably, in this process the intermediates protocatechuate, dihydroxybenzaldehyde, and vanillic acid are also detected with the yields of 18, 10, and 2 μM, respectively. After optimization of the protein expression, reaction conditions and the cell membrane permeabilization of TPA, the conversion rate achieved was as high as 79% increased by ∼157-fold. Finally, biotransformation of post-consumer PET bottle into vanillin was investigated by coupling this metabolic pathway with LCC^*WCCG*^-catalyzed PET hydrolysis (Tournier et al., 2020). Results showed that the yield of vanillin reached to 68 μM, when the two stepwise reactions finished after 24 h. Though this strategy demonstrates potentials in the biological upcycling of post-consumer plastic waste into special value-added chemicals, great attentions should be paid to further improving the lower TPA concentrations. This is believed to be the rate-limiting step for a circular and sustainable bio-PET economy.

In another study, a novel artificial microbial system was developed for efficient biodegradation of PET, which was constituted of *R. jostii*, *P. putida* and two metabolically engineered *B. subtilis* (Qi et al., 2021a). Firstly, the two-step PET-degrading metabolic pathways were achieved with two metabolically engineered *B. subtilis*. The first strain was engineered to heterologously express *Bs*_PETase, mediating the depolymerization of PET to MHET. The second strain was designed to express *Bs*_MHETase, enabling hydrolytic cleavage of MHET into TPA and EG. This functional segregation optimizes catalytic efficiency by spatially separating the sequential enzymatic reactions within specialized microbial chassis. Results demonstrated enhanced depolymerization efficiency in this dual-species microbial system, which achieved complete hydrolysis of 2 g/L BHET within 22 h, with concurrent degradation of 13.6% (weight loss) of amorphous PET films over 7 days. However, inhibitory effects from TPA and EG were also observed during BHET degradation, especially the released TPA significantly suppressed host cell proliferation. To address this limitation, *R. jostii* RHA1, a strain renowned for its robust aromatic compound catabolism, was integrated, which resulted in complete degradation of 2 g/L TPA within 28 h. This strategy effectively alleviated the TPA-mediated growth inhibition, and enabled a tripartite microbial consortium to hydrolyze 2 g/L BHET within 20 h. Especially, the optimized system achieved 31.2 ± 2.2% weight loss of PET within 7 days, representing a 17.6% enhancement. Finally, *P. putida* KT2440 was introduced with the purpose of relieving the inhibitory effect caused by EG, which is capable of transforming EG into acetaldehyde. However, it was shown that the growth of the other three bacteria would be strongly inhibited by *P. putida* when co-cultivated in nutrient-rich media. This was finally tackled with a selective medium species (inorganic salt media, W) with the addition of ammonium sulfate and potassium nitrate, and sucrose and glucose as dual carbon sources. In this way, BHET (2 g/L) could be completely degraded within 20 h catalyzed by the four-species microbial system without detectable accumulation of TPA and EG. Following optimization, the PET film demonstrated a weight loss of 7.9 ± 0.78 mg within 7 days, and the depolymerization rate reached as high as 23.2 ± 2.3%. However, the degradation rate of the four-species microbial system was lower than that obtained with the three-species system (31.2 ± 2.2%), which might be caused by the nutrient-poor medium. Overall, this study highlights a novel idea for efficient biodegradation of PET in a greener way catalyzed with the artificial microbial consortia. However, a thorny issue still needs to be resolved that is how to address the issue of achieving appropriate growth rates among different microbial strains during the co-fermentation process.

#### 3.2.3 Dual enzyme system for PET recycling

Recently, a thermophilic carboxylesterase (Est30) was engineered for efficient PET-depolymerization by relieving the inhibitory effect caused by the accumulated MHET (Zhang J. et al., 2023). It was mainly focused on (1) establishing a hydrogen-bonding network to stabilize transition-state conformations through carboxyl terminus binding, and (2) enhancing hydrophobic interactions surrounding the MHET benzene ring. Initial computational screening identified 11 out of 14 variants with improved MHET hydrolysis efficiency at 50°C (pH 7.5), among which M8 (Est30^*I*171*K/G*130*L*^), M13, and M14 (Est30^*I*171*K/M*127*S/G*130*L*^) exhibited remarkable catalytic enhancements of 36. 0-, 62. 1-, and 96.3-fold, respectively. Structural analysis demonstrated conserved active-site architecture (RMSD = 0.62 Å) and oxyanion hole geometry relative to the wild-type enzyme, while variant M14 exhibited a unique lid domain reorientation toward the hydrolase domain. Molecular dynamics (MD) simulations revealed that engineered mutations preserved critical hydrogen bonding and hydrophobic stacking interactions, demonstrating their role in transition-state stabilization. The thermostable variant M8 (T_*m*_ = 67.58°C, designated as KL-MHETase) demonstrated optimal activity under FAST-PETase-compatible conditions. Then a dual-enzyme system combining KL-MHETase and FAST-PETase (2:6 ratio) achieved 2.6-fold faster PET depolymerization and 1.64-fold higher TPA yield (99.5% purity) compared to FAST-PETase alone. However, the initial reaction kinetics revealed competitive binding between KL-MHETase and FAST-PETase, which suggests the need for spatial optimization. To address this limitation, researchers developed fusion enzymes using flexible (Gly-Ser)n linkers to connect FAST-PETase with M14 (KLS-MHETase). The engineered mutant KL36F demonstrated enhanced catalytic synergy *via* an efficient MHET channeling system, achieving 82.9% PET conversion (1.12-fold increase), 82.5% TPA yield (1.64-fold enhancement), and 99.5% product purity (1.47-fold improvement) under standard reaction conditions. Linker flexibility enabled proper orientation of active sites for direct MHET transfer, effectively preventing intermediate accumulation. At 0.35 μM loading, KL36F sustained MHET levels below inhibitory thresholds (<0.5%), while suboptimal enzyme dosing underscored the need for MHETase refinement. In industrial-scale trials (100 g/L PET), KL36F achieved >90% depolymerization efficiency, yielding 89.0% TPA with >99% purity, demonstrating robust scalability, and commercial viability. Comparative studies with other dual-enzyme systems, such as PETase/ΔBsEst and PETase/ΔChryBHETase (producing 663.1 and 617.6 μM TPA, respectively), further confirm the general efficacy of cascade enzymatic strategies. Collectively, these advancements demonstrate potential to establish sustainable plastic circularity through enzyme-driven valorization processes, while achieving operational cost reductions via streamlined downstream processing.

Similar studies further validate the efficacy of cascade enzymatic systems for PET depolymerization. Engineered BHETase variants (ΔBsEst and ΔChryBHETase) with 3.5-fold improved catalytic efficiency (*k*_*cat*_/*K*_*M*_) were incorporated into synergistic systems with PETase (Li et al., 2023a). The PETase/ΔBsEst and PETase/ΔChryBHETase combinations demonstrated exceptional performance, producing 663.1 and 617.6 μM TPA within 24 h, which represents a 7.0- and 6.5-fold increases, respectively over PETase monotherapy (95 μM). This enhancement mechanism parallels the KL36F system, where coordinated actions between PET-degrading and intermediate-processing enzymes prevents metabolic bottlenecks. The observed TPA yields (82.5%–89.0%) and purities (>99%) across different dual-enzyme systems further highlight its generalizability for industrial-scale plastic valorization. These findings corroborate the broader applicability of dual-enzyme cascades in establishing closed-loop PET recycling paradigms.

Recent advancements in enzymatic PET degradation have also led to the development of an innovative dual-enzyme system that operates through signal peptide-independent secretion (Aer et al., 2024b). This system employs a bifunctional chimeric enzyme (designated TfH-FPE), which is created by fusing FAST-PETase with TfH, a phospholipase capable of modifying cell membrane permeability. The TfH component facilitates extracellular secretion of the fusion enzyme while simultaneously enhancing substrate accessibility through membrane modulation. Results showed that this synergistic design significantly improves the bio-depolymerization efficiency of PET compared to single-enzyme approaches. The engineered TfH-FPE chimeric enzyme achieved a secretion yield of 104 ± 5.2 mg/L under optimized conditions, representing a 32.5-fold enhancement over conventional signal peptide-dependent systems. The enhanced secretory efficiency directly underpinned superior PET depolymerization efficacy, with TfH-FPE demonstrating sixfold increased product titer relative to standalone FAST-PETase and twofold elevated yield vs. equimolar discrete enzyme cocktails under identical operational parameters. In addition, kinetic characterization revealed substantial functional improvements in the fusion enzyme, including a 4.7-fold increase in hydrolysis rate constant (*k*_*cat*_/*K*_*m*_) and 4.1-fold enhanced substrate adsorption affinity toward PET films at 50°C. Time-course analyses demonstrated temperature-dependent degradation profiles, and the product accumulation reached saturation (8.9 ± 0.3 mM) within 48 h at 50°C, while exhibiting sustained linear release kinetics over 7 days at 40°C. Notably, despite showing 1.5-fold greater cumulative product yields than FAST-PETase at 40°C, TfH-FPE displayed counterbalanced kinetic properties with a threefold reduction in hydrolysis rate but 4.1-fold higher PET binding affinity. The decoupled catalytic kinetics and substrate adsorption profiles reveal the bifunctional merits of the fusion architecture, including prolonged operational stability through strengthened substrate binding, and the attenuated product inhibition achieved *via* spatial segregation of hydrolytic domains.

Moreover, the TfH-FPE fusion enzyme also exhibited superior PET depolymerization kinetics across all experimental conditions, which is capable of achieving maximal efficiency at 50°C with 12.2 ± 0.9 mM product release, a twofold greater than equimolar mixtures of standalone FAST-PETase and TfH. This might be mechanistically linked to enzyme colocalization effects, where spatial proximity between the phospholipase and PETase domains minimizes substrate diffusion barriers and enables cooperative catalysis. Importantly, this study establishes two paradigm-shifting innovations in enzymatic PET recycling. The autonomous secretion framework, a signal peptide-independent secretory mechanism, would enable efficient extracellular delivery of multi-domain biocatalysts (104 ± 5.2 mg/L yield). The synergistic interfacial catalysis mechanism integrates TfH-driven membrane permeabilization for enhanced substrate accessibility with FAST-PETase-mediated processive hydrolysis, which would establish a dual-functional cascade that optimizes both catalytic initiation and chain-scission efficiency. This tandem action achieved 4.7-fold faster PET film deconstruction compared to monofunctional systems. Collectively, these advantages position the TfH-FPE system as a paradigmatic framework for next-generation biocatalyst design: (1) autonomous platforms circumventing upstream protein engineering constraints through self-sufficient catalysis; (2) spatially optimized cascade architectures bypassing diffusion barriers *via* enzyme colocalization; and (3) thermostable systems preserving functional integrity across industrial processing temperatures (40–50°C).

The integration of PETase and MHETase into dual-enzyme systems effectively mitigates intermediate product inhibition (e.g., MHET accumulation), thereby achieving significantly enhanced PET degradation efficiency through synergistic catalytic action. In contrast, single-enzyme systems (e.g., free LCC^*ICCG*^) usually required exogenous MHETase supplementation to alleviate inhibition, which might complicate the process design. Dual-enzyme consortia outperformed single systems by 50-fold in PET-to-TPA conversion due to synchronized depolymerization and intermediate metabolism (Diao et al., 2023).

### 3.3 Metabolic engineering of *Yarrowia lipolytica*

*Yarrowia lipolytica* (Cao et al., 2023; Park and Ledesma-Amaro, 2023; Liu Y. et al., 2024) is of great significance in the biotech field as it can efficiently produce a variety of valuable metabolites like lipids and polyols, and has been widely applied in the food, cosmetic and biofuel industries. As a premier microbial model, it enables mechanistic dissection of metabolic networks and cellular dynamics, serving as a translational platform bridging basic research in core biological principles with applied innovations in industrial biotechnology.

#### 3.3.1 Direct heterologous expression of PET-degrading enzymes in *Y. lipolytica*

Recent advances have demonstrated successful heterologous expression of a codon-optimized PETase gene in *Y. lipolytica* for direct PET degradation (Kosiorowska et al., 2022b). The PETase coding sequence (GenBank: GAP38373.1) was codon-optimized for yeast expression and placed under the transcriptional control of the native XPR2 secretion signal to facilitate enzyme extracellular secretion. The optimized expression cassette was subsequently integrated into the genome of *Y. lipolytica* strain AJD2, generating the engineered strain AJD 2 pAD PET_IS. Functional characterization revealed successful secretory production of active PETase without causing significant growth defects. However, during PET degradation assays, both the engineered strain and AJD2 control failed to assimilate TPA monomers, and this contrasted with previous observations by Costa et al. (2020), where the *Y. lipolytica* derivatives demonstrated TPA utilization. This phenotypic discrepancy may be attributed to differences in cultivation conditions or strain-specific metabolic capabilities. Notably, both strains exhibited EG assimilation capacity, and the engineered AJD 2 pAD PET_IS strain achieved complete hydrolysis of MHET to TPA within 96 h. When applied to PET degradation, TPA accumulation became detectable in AJD 2 pAD PET_IS culture supernatants for 120 h, whereas neither TPA nor BHET were observed in AJD2 controls. These metabolic profiles differ significantly from the TPA utilization patterns reported by Costa et al. (2020), which suggests potential strain-specific differences in aromatic compound metabolism or variations in experimental conditions. Further optimization revealed that supplementation with specific metal ions markedly enhanced PET depolymerization efficiency. The addition of 2.5 mM MnSO_4_ and 1 mM CuSO_4_ increased TPA yields to 75 mg⋅L^–1^ (7.5-fold enhancement) and 62 mg⋅L^–1^ (6.2-fold enhancement), respectively. Most remarkably, incorporation of 1% (w/v) olive oil into the culture medium achieved a maximum TPA of 124 mg⋅L^–1^. This synergistic effect likely stems from the dual function of olive oil as both a hydrophobic surface-modifying agent and an inducer of endogenous lipase production, which may collaboratively enhance PET accessibility and enzymatic hydrolysis through interfacial activation mechanisms. Intriguingly, cultivation in 0.3-L Erlenmeyer flasks revealed growth inhibitory effects associated with salt supplementation, which could be potentially linked to suboptimal oxygen transfer efficiency and altered cellular proliferation kinetics. This contrasts with improved biomass accumulation observed in scaled bioreactor systems, where enhanced aeration and nutrient distribution likely mitigated the ionic stress effects documented in flask-scale experiments. Notably, olive oil supplementation induced pronounced physicochemical changes in PET substrates, including the accelerated surface corrosion, and substantial polymer mass reduction (53.05% ± 2.1% of initial mass). These alterations correlated with enhanced biodegradation efficiency, potentially through amphipathic compound-mediated biofilm formation and increased polymer surface hydrophilicity. This integrated approach demonstrates substantial potential for synergistic PET bioremediation during yeast cultivation, where microbial proliferation and enzymatic depolymerization proceed concurrently. The scale-dependent parameter optimization highlights the necessity for bioreactor-specific condition tailoring to maximize degradation efficiency while maintaining culture viability.

#### 3.3.2 Metabolic engineering and fermentation engineering of *Y. lipolytica* for PET-cycling

**FIGURE 12:**
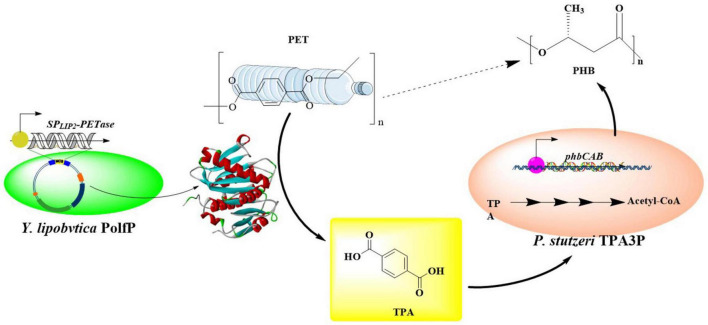
Pictorial representation of PET bioconversion *via* the co-cultivation of engineered PETase- and PHB-producing microorganisms.

Recently, a novel co-cultivation system for the upcycling of PET waste was developed, which is capable of PET-degradation and polyhydroxybutyrate (PHB) biosynthesis (Figure 12; Liu et al., 2021). *Y. lipolytica* Po1f was firstly engineered to express *Is*PETase. The system achieved signal peptide-free secretion of functional *Is*PETase through innovative utilization of SP_*LIP*2_, a truncated secretory module derived from the Lip2-encoded extracellular lipase. Proteomic characterization revealed this hybrid secretion system enhances PETase yields by 3.8-fold compared to conventional signal peptide-dependent approaches, which establishes SPLIP2 as a universal cargo-transport scaffold for yeast-based biocatalyst production. This bifunctional design overcomes traditional bottlenecks in secretory pathway engineering, enabling continuous PET hydrolysis (≥72 h) with 92% monomer recovery efficiency from post-consumer waste streams. Then, a TPA-degrading strain *Pseudomonas stutzeri* TPA3 was employed for the biosynthesis of PHB, which was achieved by the integration of the *phbCAB* operon from *Ralstonia eutropha*. It was confirmed that the resulting *P. stutzeri* TPA3P was capable of bio-synthesizing PHB, and 11.56 wt% of cell dry weight PHB could be accumulated from 10 g/L TPA as the sole carbon source. Co-cultivation of *P. stutzeri* TPA3P and *Y. lipolytica* Po1f with glucose and BHET as carbon sources enables the direct conversion of BHET to PHB, achieving 5.16 g/L BHET hydrolysis in 12 h and 3.66% PHB accumulation in 54 h. In addition, the biomass of *P. stutzeri* TPA3P was shown to experience a significant decrease toward the end of the cultivation, which was likely caused by a deficiency in TPA or other unidentified factors. Though, the ultimate yield of PHB did not meet expectations (due to the low efficiency of *Is*PETase), this approach offers a promising strategy for PET waste biodegradation and upcycling using engineered microorganisms.

**FIGURE 13:**
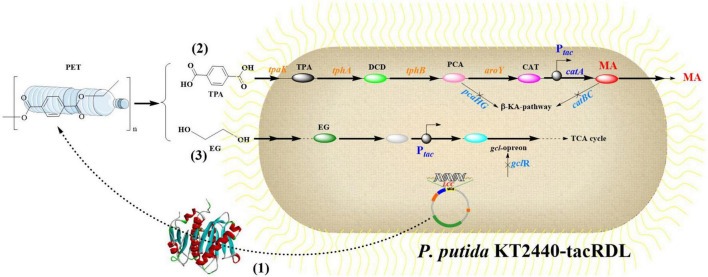
Graphic illustration of the continuous conversion of PET to muconic acid (MA) with the engineered *P. putida* KT2440.

To address the issue of low reaction efficiency caused by *Is*PETase, a novel strategy has been proposed that facilitates the continuous conversion of PET to muconic acid (MA) with an engineered strain of *P. putida* KT2440 (Figure 13; Liu et al., 2022). Firstly, the *pcaHG* gene (encoding the protocatechuate 3,4-dioxygenase) was replaced by the *tph* cluster to enhance the utilization of TPA and inhibit the metabolic pathway of protocatechuate. Then *aroY* gene (encoding protocatechuate decarboxylase) and *ecdB* gene (encoding the flavin prenyltransferase) were integrated for the catalytic conversion of protocatechuate to catechol. In addition, MA metabolic pathway was further optimized by deletion of the downstream *catBC* to effectively halt the subsequent β-ketoadipate (β-KA) pathway. The engineered strain *P. putida* KT2440-tac demonstrates the capability to synthesize 4.63 mM of *cis*-MA from 12.86 mM TPA. However, it is noteworthy that accumulated protocatechuate was also observed during this process. To relieve the inhibition caused by the *gclR* regulator, deletion of the *gclR* and overexpression of the glycolate oxidase gene (*glcDEF*) under the control of *P*_*tac*_ were successfully achieved. The resulting *P. putida* KT2440-tacRD was further engineered to express PET hydrolase LCC, and the obtained host KT2440-tacRDL was capable of PET-degradation and growing on EG simultaneously. The crude LCC, expressed by *P. putida* KT2440-tacRDL, was capable of catalyzing minimal accumulation of BHET (<0.23 mM). As anticipated, the concentration of MHET exhibited an initial increase followed by a gradual decline, culminating in the production of 43.66 mM TPA at a rate of 0.15 g/(L⋅h) within a 10 ml reaction system. Under the optimized condition, *P. putida* KT2440-tacRDL is able to produce 32.33 mM MA at a rate of 0.54 mmol/(L⋅h). Notably, EG metabolism appears to be markedly suppressed in LB medium, a phenomenon initially hypothesized to stem from β-ketoadipic acid accumulation based on prior metabolic engineering studies (Werner et al., 2021). However, experimental evidence from β-ketoadipate (β-KA) pathway knockout models revealed persistent metabolic inhibition, which strongly suggested the existence of alternative regulatory mechanisms beyond this pathway. This paradoxical observation underscores the necessity for systematic investigations into the multifaceted regulatory network governing EG catabolism, particularly focusing on potential cross-talk between central metabolic pathways and quorum-sensing systems in microbial consortia.

Recently, a groundbreaking biocatalytic strategy has also been developed for direct PET degradation through metabolic engineering of *Y. lipolytica* (Kosiorowska et al., 2022a; Janek et al., 2020). This system combines extracellular expression of lipase (YlLip2) with *F. solani*-derived cutinase (FsC) in a synergistic enzymatic approach. The initial optimization involved overexpressing YlLip2 in *Y. lipolytica* A101 using a high-efficiency hybrid promoter (UAS1B16-TEF). To maximize enzyme yield, genomic modifications were implemented through deletion of two native proteases (AEPp and AXpp), yielding *Y. lipolytica* AJD ΔXΔA-Lip2. Biochemical characterization revealed optimal YlLip2 exhibits maximum enzymatic activity under optimal conditions of 37°C and pH 8.0. Notably, the enzyme demonstrated exceptional tolerance to lipopeptide biosurfactants (amphisin and viscosinamide), and enhancements of 160% and 200% could be achieved at 0.2 and 0.15 mM concentrations, respectively. This positive effect appears to involve surfactant-mediated enhancement of substrate-enzyme interactions at the catalytic site. Based on these findings, FsC was further overexpressed resulting in the host *Y. lipolytica* AJD 2 pAD CUT_FS for the metabolism of PET. With this host cell, increased levels of degradation products (e.g., TPA and MHET) were achieved, reaching 1.51 and 0.45 g⋅L^–1^, respectively, after 240 h in a 1 L bioreactor. Although, bioprocess scaling demonstrated remarkable scalability, the PET degradation efficiency quantified (the TPA to MHET ratio) in various culture systems was found to be different. It was 0.55:1 in deep well plates (DWP), rose to 2.95:1 in flask cultures, and impressively peaked at 4.63:1 in bioreactor cultures. The progressive increase in TPA dominance correlates with the biomass-dependent cutinase production dynamics (Vertommen et al., 2005). Scanning electron microscopy confirmed substantial surface erosion of PET films, which could provide visual validation of degradation efficacy. Additionally, systematic screening identified critical media components capable of enhancing PET depolymerization in DWP cultures. A total of 2.5 mM calcium chloride (CaCl_2_) could maximize TPA release and minimize MHET release, when combined with 2.5 mM MnSO_4_ and 2.5 mM MgSO_4_. Moreover, 2.5 mM MnSO_4_ and MgSO_4_ alone could significantly reduce the MHET levels. This engineered biocatalytic platform demonstrates substantial potential for industrial PET biorecycling applications, combining genetic engineering optimization with bioprocess parameter tuning to achieve enhanced polymer breakdown efficiency.

#### 3.3.3 Solid-state fermentation of *Y. lipolytica* for PET depolymerization

In a recent study, the potential of *Y. lipolytica* for PET depolymerization was systematically investigated under both solid-state fermentation (SSF) and submerged cultivation conditions (Sales et al., 2021; Sales et al., 2022). The efficacy of three low-cost inducers (apple peels, commercial cork, and PET) was evaluated for enhancing lipase and esterase production during SSF of soybean bran by *Y. lipolytica*. It was shown that addition of 5 or 20 wt% of commercial cork would result in a 16% increase in lipase activity and a remarkable 131% increase in esterase activity, respectively. In addition, the incorporation of PET into the fermentation process also significantly boosted the esterase activity of the enzymatic extracts by up to 69%. Notably, enzymatic extracts derived from fermentation samples supplemented with 20 wt% PET and 20 wt% apple peels could achieve the highest concentration of TPA (21.2 μM) after 1 week. Meanwhile, enzymes from cork-enriched SSF demonstrated superior efficiency in hydrolyzing BHET, a key PET degradation intermediate. In addition to the biocatalysts produced by *Y. lipolytica* through SSF, the lipase B from *Candida antarctica* (CALB) was also added in this PET-degradation process. It was shown that the enzymes were capable of hydrolyzing BHET, producing TPA (131.31 M), and PET, leading to a TPA concentration of 42.80 M after 168 h.

Submerged cultivation in YT medium (1% yeast extract, 2% tryptone) achieved a TPA concentration of 65.40 μmol L^–1^ after 96 h. Tryptone-containing media (e.g., YT and YTD) would also significantly enhance the secretion of PET-hydrolytic enzymes. When scaling to a 0.5-L benchtop bioreactor, yield of TPA could be further improved to 94.31 μmol L^–1^, which is 121% higher than SSF-derived results. While these results highlight the promise of *Y. lipolytica*-derived biocatalysts in PET depolymerization, critical gaps still remain including: (1) mechanistic insights into enzymatic PET hydrolysis; (2) optimization of biosurfactant and catalytic protein production; (3) profiling of microbial cell adhesion and PET monomer assimilation; and (4) promising strategies to sustain enzymatic activity and cell viability during prolonged degradation.

Recent advances in metabolic engineering have enabled the development of optimized biodegradation pathways for PET through systematic comparison of prokaryotic and eukaryotic enzymatic systems. For prokaryotic systems, *P. putida* chassis strains demonstrate remarkable PET-hydrolyzing capabilities through engineered secretion systems of *Is* PETase enzymes. Conversely, eukaryotic platforms like *Blastobotrys* spp. offer distinct advantages in post-translational modification and compartmentalized expression of PET-depolymerizing enzymes, particularly when utilizing hybrid pathways combining fungal cutinases with yeast secretory mechanisms. This dichotomy highlights the complementary potentials of microbials in developing consolidated bioprocesses for PET waste valorization. Especially, the glycolytic pathway of *P. putida* efficiently channels EG into the TCA cycle *via* glyoxylate shunt catalysis, which would lead to complete mineralization. However, *Blastobotrys* spp. employ alternative PCA decarboxylation pathways but lack robust glyoxylate shunt activity, which might result in limited EG utilization. Collectively, the modular metabolic networks and solvent tolerance make *P. putida* as a superior chassis for PET upcycling, whereas eukaryotic systems might be less developed for large-scale applications.

### 3.4 Rational design of enzyme-PET interface engineering

Immobilization of PETase enzymes significantly enhances the efficiency of PET biodegradation, as it allows the enzymes to be reused multiple times and maintain their activity under various conditions (Schwaminger et al., 2021). Moreover, it also plays a crucial role in the practical application of PET biodegradation, enabling more stable and efficient degradation processes in industrial settings and contributing to the reduction of environmental pollution caused by PET waste.

A breakthrough in enzymatic plastic degradation has been achieved through the development of MC@CaZn-MOF, a hierarchically structured biocatalytic composite engineered for *in situ* PET surface remodeling (Zhang et al., 2024). The MOF-based system integrates three key innovations, including the chemoselective immobilization of cutinase variants, the surface-adaptive topology, and the dynamic hydration regulation. The protein engineering strategy involved two key modifications to optimize enzymatic performance. Initially, the hydrophobin (HFBI), a self-assembling interfacial protein, was genetically fused to the N-terminus of *Is*PETase through a flexible (G4S)2 linker, creating the chimeric protein HFBI-*Is*PETase. To enable modular assembly, the SpyCatcher/SpyTag conjugation system was incorporated by fusing SpyCatcher to the C-terminus of HFBI-*Is*PETase and SpyTag to MHETase, yielding HFBI-*Is*PETase-SpyCatcher and MHETase-SpyTag constructs respectively. This covalent peptide-protein ligation system facilitates irreversible molecular coupling through spontaneous isopeptide bond formation between SpyCatcher and SpyTag. Through SpyC/SpyT-mediated self-assembly, a multienzyme complex (designated HPCTM) was successfully constructed, which synergistically combines surface-active HFBI domains with complementary enzymatic activities. Biochemical characterization revealed that HPCTM exhibited enhanced BHET hydrolysis efficiency surpassing both free PETase and the PETase + MHETase combination. Moreover, improved interfacial properties were evidenced by a 39° reduction in the WCA of PET surface (from 84.2° to 45.1°), and superior PET degradation performance was achieved with 27.3% weight loss (1.6-fold improvement). Additionally, efficient metabolic channeling resulted in the release of 253 μM TPA while maintaining a low MHET intermediate level (20 μM). Notably, the HFBI-mediated surface localization combined with enzyme colocalization through SpyC/SpyT conjugation created a catalytic nanoenvironment that simultaneously enhancing substrate accessibility, promoting intermediate transfer, and minimizing product inhibition.

Then HPCTM was immobilized onto the nanoparticles CaZn-MOF forming the biomimetic module (MC@CaZn-MOF) with a Zn: Ca ratio of 2:3 and HPCTM concentration of 0.6 mg/ml. MC@CaZn-MOF displayed an improved BHET-hydrolyzing activity with 340 μM TPA and 22.5 μM MHET produced after 12 h, and the WCA could be further decreased to 47°. Notably, MC@CaZn-MOF was capable of directly breaking down untreated AGf-PET, and the weight loss (96%) was 6.3-fold higher than that of HPCTM after 5 days. Especially, it is also capable of efficiently breaking down PET bottles, with a maximum release of 9.2 mM PET monomers after 5 days. The material demonstrated exceptional long-term durability, thermal stability, extended storage stability under ambient conditions, and remarkable recyclability over multiple cycles without significant performance degradation. This groundbreaking platform technology pioneers a novel paradigm for integrated cascade enzymatic depolymerization and microbial bioassimilation systems, showing particular efficacy in handling complex mixed-plastic waste streams under non-sterile environmental conditions.

### 3.5 Metabolic engineering of microalgal for enhanced PET-degradation

Recent advances have demonstrated the potential of green microalgal chloroplast systems for sustainable bioproduction of *Is*PETase and plastic biodegradation applications (Di Rocco et al., 2023). A pioneering approach involves constructing a synthetic *Is*PETase gene cassette incorporating three critical components: (1) codon-optimized sequences tailored for chloroplast expression, (2) an N-terminal Sec-type signal peptide to facilitate protein translocation across thylakoid membranes, and (3) the *psbH* photosynthesis gene as a dual-functional element serving both as a selection marker and photosynthetic restoration module. This engineered construct was cloned into the pSRSapI vector, and subsequently introduced into *Chlamydomonas reinhardtii* strain TN72 at a neutral locus between *psbH* and *trnE2*, restoring the wild-type copy of *psbH*. The optimized design achieved two pivotal advancements: (1) the *psbH* marker enabled chloroplast transformation selection *via* phototrophic restoration, eliminating reliance on antibiotic resistance genes; (2) the signal peptide directed the enzyme translocation to the thylakoid lumen-an oxidizing subcellular environment that promotes proper disulfide bond formation. Then, SDS-PAGE analysis and tandem mass spectrometry of cell extracts from the TN72:PETase.1 cell line confirmed the presence of PETase. Especially, atomic force microscopy (AFM) imaging showed that the enzyme is capable of inducing morphological changes, such as the formation of holes, on both PET and PCP samples. This was also confirmed by the quantitative analysis with a decrease in surface correlation length (ξ, from 2.2 ± 0.6 to 0.22 ± 0.08 μm for PET film, and from 0.53 ± 0.1 to 0.21 ± 0.03 μm for PCP) and an increase in surface roughness (Δσ_*rmsPCP*_ = + 17.5 nm) for PCP. This might result from the fact that the technical-grade PET foil is more resilient to stress than postconsumer plastic foil. The PETase activity was further verified by the digestion of PET powder and BHET, which yielded 0.02 mM TPA and 0.8 mM monomer products, respectively. However, for PCP only 0.20 mM BHET was detected without TPA, which might be caused by the low reaction temperature (30°C) and a relatively short catalyzing period.

In this study, a platform for producing PETase in the chloroplast of *C. reinhardtii* was successfully established. Although the recombinant protein yield was lower than that of bacterial systems, the activity of the algal-expressed enzyme in terms of TPA release was in line with published data for the wild-type protein. This paves the way for future studies focused on producing improved PETase variants and other plastics-degrading enzymes to combat marine plastic pollution in this sustainable platform. Especially, the microalgae offer several advantages for PETase production, such as reduced scale-up costs, and the ability to precisely target transgenic DNA into the plastome *via* homologous recombination.

### 3.6 Promising strategy for the development of novel PET hydrolase

#### 3.6.1 Development of novel high-throughput screening strategies

To address the limitations of existing methods in developing potential PETase mutants, a revolutionary platform has been developed, which is capable of simultaneously evaluating large PET hydrolase libraries (10^4^–10^5^ variants) for protein solubility, thermostability, and catalytic activity (Groseclose et al., 2024). It uses plate-based split green fluorescent protein assays and model substrate screens. Generally, this system is composed of four main parts: (1) generation of a vast library of variants through DNA shuffling; (2) establishment of high-throughput screening to test thousands of variants for activity, expression, and solubility on BHET substrates; (3) validation of a smaller subset of these variants using cell lysates and different PET substrates for improved hydrolyzing activity; and (4) characterization of the top variants using purified enzymes against various PET substrates. Each evolution cycle lasts about 6–8 weeks, and the identified variants would be used in subsequent rounds. After multiple cycles, the final variants are characterized for catalytic performance using HPLC and bioreactors. For the initial high-throughput co-screening, genetically tagged variants with GFP11 were evaluated on BHET model substrate agar plates *via* split GFP complementation. Enzyme libraries were cultivated on membranes overnight, induced with IPTG, then transferred to BHET plates for partial lysis. Plates were incubated at 65–70°C for 5–20 h. The integration of BHET hydrolysis with split-GFP complementation assays facilitated high-throughput screening of colonies exhibiting enhanced enzymatic activity, improved protein solubility, and stable expression profiles, enabling efficient pre-identification of optimal variants for subsequent industrial applications. Coarse screening focused on colonies with larger clearing zones or brighter green fluorescence, which are followed by fine screening in 96-well plates for thermostability. Promising variants were further validated on PET substrates. Throughout, selection pressures were incrementally increased (e.g., BHET concentrations, temperatures, and durations) to develop optimal mutants.

This platform was used to enhance the catalytic activity of wild-type LCC (LCC-WT). After two evolution cycles, LCC-F2 (LCC^*V*118*I/A*149*V/L*159*E/V*202*I*^) showed higher activity and comparable solubility on BHET agar plates. For high-crystallinity PET powder degradation, LCC-F2 was 13% more active than LCC but 23% less active than LCC^*ICCG*^. After the third cycle with increased BHET and temperature, LCC-F6 (LCC^*P*38*L/L*117*P/A*149*V/Y*127*G/D*238*C/F*243*I/S*283*C*^) matched LCC^*ICCG*^ on both PET substrates. The fourth cycle yielded LCC-B8 and LCC-C9, which significantly outperformed LCC^*ICCG*^ on amorphous PET film but were less active on high-crystallinity PET powder. To improve thermostability, an additional evolution cycle with preheat treatment and increased BHET concentration was conducted, resulting in LCC-LANL (LCC^*Y*127*G/D*238*C/F*243*I/S*283*C/P*38*L/Y*61*C/M*91*I/L*117*P/A*149*V/H*218*Y*/*Q*224*H*/*S*247*L/T*256*I*^). It showed a 14.3-fold increase in aromatic product release and a 13.9-fold increase in maximal rate on amorphous PET film compared to LCC^*ICCG*^. At 68°C, the initial rate of LCC-LANL was 1.21 g⋅L^–1^⋅h^–1^ TPA and 1.77 g⋅L^–1^⋅h^–1^ aromatic products. However, its activity declined after 8 h at 70°C, possibly due to decreased thermostability or product inhibition. This is particularly caused by the S247L mutation which boosts the hydrophobic surface area around the substrate-binding site, therefore benefiting catalysis in the presence of hydrophobic substrates. Additionally, mutations like M91I and T256I might also contribute to a more uniform hydrophobic core, which, despite creating some cavities, could increase the mobility and catalytic efficiency at lower temperatures.

Structural characterization demonstrated that specific mutations in the LCC-LANL variant (including S247L, M91I, and T256I) significantly improved catalytic performance on PET substrates, though these alterations appeared to reduce thermal stability compared to the parental enzyme configuration. Docking simulations showed that LCC-LANL has more productive binding modes for PET substrates than LCC^*ICCG*^, which contributes to its improved catalytic efficiency on amorphous PET films. However, this trend was not observed for amorphous PET powder, and this suggests that milling may affect enzyme-substrate interactions. Overall, while LCC-LANL demonstrated significant improvements in catalytic efficiency on amorphous PET film, its thermostability and performance at elevated temperatures or over extended periods remain limitations for industrial applications.

A novel high-throughput fluorescence-based screening platform for PET hydrolases was successfully established, demonstrating robust performance with a coefficient of variation (CV) of 17.3% and Z factor exceeding 0.7 (Shi et al., 2023). This dual-fluorescence detection system integrates mCarmine-labeled enzyme quantification (Ex/Em 603/675 nm) with fluorescein-based substrate hydrolysis monitoring (Ex/Em 494/525 nm) in 96-well plate format. The methodology enables simultaneous measurement of enzyme concentration and catalytic activity through wavelength-resolved fluorescence detection, effectively eliminating signal interference between the two fluorophores. Using this method, about 10,000 clones were screened in three rounds of directed evolution, leading to the discovery of the promising PETase M5 variant (DepoPETase^*T*88*I/D*186*H/D*220*N/N*233*K/N*246*D/R*260*Y/S*290*P*^). Its catalytic activity toward BHET-OH increased 1.9-fold, and M5 is capable of retaining 97.3% of its initial activity at 50°C for 30 min. When depolymerizing gf-PET with M5 at 50°C, complete degradation occurred in 48 h. After pH adjustment, 14.07 mM of products (TPA and MHET) were released. The PET-degrading performance of M5 was similar to FAST-PETase initially, but surpassed it after 24 h, releasing 16.6% more products. DepoPETase effectively depolymerized various untreated post-consumer PET waste in 1.5–4.5 days, showing potentials for recycling 10%-crystallinity PET materials. However, higher-crystallinity PET needs pre-treatment for better enzymatic depolymerization. In a scaled-up process, DepoPETase depolymerized 19.10 g of pc-PET film in a 3 L bioreactor at 50°C in 120 h (0.4% W_*enzyme*_/W_*PET*_, enzyme loading). The reaction exhibited a rapid initial rate of 0.5 mM per hour for both TPA and MHET, with approximately 70% of the total products being liberated within the initial 48 h. The subsequent decline in catalytic efficiency was likely attributed to product inhibition mechanisms, which highlighted the critical importance of maintaining optimal pH conditions to preserve enzyme structural integrity and maximize catalytic performance.

#### 3.6.2 Surface charge engineering of the PET degrading enzymes

In a recent study, 24 fungal cutinases were heterogeneously expressed, in which TaC from *Thermocarpiscus australiensis* was particularly effective on PET (Brinch-Pedersen et al., 2024). It displayed up to fourfold higher catalytic activity and greater thermal stability than HiC (Hong et al., 2019). However, the requirement for elevated salt concentrations [primarily attributed to electrostatic repulsion (Ribitsch et al., 2017)] to achieve optimal catalytic activity significantly limits its practical applicability in industrial settings. To solve this, surface charge engineering was explored by creating enzyme variants with reduced negative charge. Forty TaC variants with decreased negative surface charge were designed and generated. This was mainly achieved by neutralizing acidic residues, introducing basic residues through isosteric replacements, co-evolutionary energy-based selections, and utilization of structural alignments with HiC for successful residue introductions. Six mutants were identified (TaC^*D*41*A*^, TaC^*E*68*G*^, TaCD82A, TaC^*D*41*A/E*68*A*^, TaC^*D*41*A/D*82*A*^, and TaC^*E*68*G/D*82*A*^) with twofold or higher activity and increased PET adsorption with low salt dependence. Notably, TaC^*E*68*G*^ and TaC^*E*68*G/D*82*A*^ had catalytic activity matching or exceeding wild-type TaC under 500 mM NaCl, which is able to eliminate the salt requirement while maintaining efficiency. At 60°C after 24 h, the most efficient variant, TaC^*D*41*A/E*68*G*^, degraded about 4.5% of highly recalcitrant PET powder, more than wild TaC (less than 1%) and HiC (less than 2%). Given these, the surface charge engineering strategy is expected to aid in developing PET hydrolases with balanced substrate-surface affinity.

Similarly, it was found that the cationic surfactant (C_12_-N(CH_3_)^3+^) could significantly improve (increased by 12.7-fold) the activity of the PET hydrolase, TfCut2, which possessed a negative net charge under the experimental pH conditions (Furukawa et al., 2019). In addition, the cationic surfactant (cetyltrimethylammonium bromide, CTAB) could enhance the catalytic performance of TfC (with a net negative charge, increased by fivefold for the turnover rate) and LCC (with a net positive charge). This is estimated to result from the cationic nature of CTAB, which would interact with the negatively charged enzymes and be capable of probably altering the conformation and increasing the affinity for the PET substrate (Arnling Bååth et al., 2022). It was also reported that introduction of the basic amino acid residues (the positively charged surface residues) to the PET hydrolase PET2 would also increase the binding rate constant (PET2 7M, increased by 2.7-fold) and lead to higher catalytic activity (increased by 6.8-fold) (Nakamura et al., 2021).

#### 3.6.3 Development of the surface crowding model

Previous research reported that *Is*PETase shows enzyme-concentration- dependent inhibition, which is possibly caused by surface crowding (Avilan et al., 2023; Skóra et al., 2021). Given this, in a recent study, wild *Is*PETase and the thermostable TS-PETase variant (*Is*PETase^*S*121*E/D*186*H/N*233*C/R*280*A*/*S*282*C*^) were further engineered with a kinetic surface crowding model for improved catalytic performance and decreased crowding tendency (Zhong-Johnson et al., 2024). Directed evolution of *Is*PETase and TS-PETase resulted in a novel mutant, T116P. When introduced into TS-PETase (forming TSP-PETase), it increased the maximum product accumulation rate by 30%. The additionally stepwise incorporation of the S238N and S290P mutation into TSP-PETase could all improve the thermostability of the resulting mutants by ∼1.5°C. However, this modification would lead to slightly reduced peak productivity of the engineered mutants. The decreased activity was estimated to result in the surface crowding during degradation process of PET films. Fluorescence techniques further confirmed that *Is*PETase experiences crowded conditions similar to those induced by macromolecular crowding agents. MD simulations also revealed crowding would reduce enzyme flexibility and inhibit the formation of productive active sites, especially in substrate-bound enzymes. Although active-site flexibility was less affected, crowding led to enzyme aggregation and decreased mobility. These results were further confirmed by the findings that activity of *Is*PETase decreased with increasing Ficoll 70 concentration (200–400 mg/ml), and the decreased BHET conversion matched simulation results.

This model well-illustrated the higher productivity of evolved variants TSP and TSPNP, which showed significant crowding effects on catalytic activity. Especially, sensitivity analysis emphasized the importance of binding capacity (Γ) and the catalytic rate (*k*_*cat*_, *uc*) while reducing the crowding effect (*K*_*c*_) for maximum productivity. The model also demonstrated that increased temperatures could boost TS-PETase variant productivity by enhancing enzyme binding and catalytic rates, without the low-temperature crowding inhibition.

The surface-crowding model, which focuses on enzymes transitioning to a packed state upon adsorption, explains the decrease in catalytic efficiency in crowded environments. It is crucial for understanding enzyme behavior under such conditions. Especially, it highlights crowding behavior as a key factor in enzyme engineering for enhanced PET degradation.

#### 3.6.4 Ancestral protein reconstruction

To date, diverse protein engineering strategies including random mutagenesis, machine learning, and rational design have been successfully implemented to enhance PET-degrading enzymes. Primary optimization targets typically involve substrate-binding site engineering, modulation of the conserved tryptophan residue (“wobbling Trp”), and residues proximal to catalytic disulfide bonds. These approaches have yielded improved enzyme variants such as LCC^*ICCG*^, DuraPETase, ThermoPETase, HotPETase, and FAST-PETas.

In addition, integration of ASR has become an indispensable approach in computational enzyme design, which would offer unprecedented capabilities to elucidate evolutionary trajectories of protein families and systematically identify functionally critical distal mutations that evade traditional structure-based rational design (Thomson et al., 2022; Hu et al., 2023; VanAntwerp et al., 2022). This methodology not only facilitates the resurrection of ancestral enzymatic properties like thermostability and conformational plasticity, but also enables targeted engineering of allosteric networks through evolutionary-guided modifications at spatially distant regulatory sites. This methodology involves the following main steps: (1) phylogenetic reconstruction, (2) ancestral sequence inference, and (3) computational identification of stabilizing mutations. For example, the integration of ASR with computational tools like Rosetta-based PROSS (Protein Repair One-Stop Shop) (Goldenzweig et al., 2016) facilitates systematic exploration of sequence space for stability-enhancing mutations.

A landmark ASR application was demonstrated in the engineering of *Is*PETase (Joho et al., 2023). Researchers reconstructed ancestral enzymes using GRASP and CodeML algorithms, which revealed the evolutionary divergence of *Is*PETase from actinobacterial cutinases. Concurrently, PROSS-guided engineering introduced 6–27 mutations per variant, strategically optimizing structural stability while preserving the catalytic triad (Ser160-Asp206-His237) and active-site architecture critical for substrate binding. Among 15 engineered variants, ancestral and PROSS-derived enzymes exhibited up to 20°C increases in melting temperature (T_*m*_), with GrAnc8 achieving optimal activity-stability balance at elevated temperatures. The evolutionary trajectory of PET-degrading enzymes reveals that ancestral cutinases exhibited limited substrate promiscuity toward synthetic polyesters, indicating a functional specialization event coinciding with the environmental proliferation of PET plastics since their industrial-scale production.

Structural analyses highlighted key determinants of thermostability and activity. A disulfide bridge in GrAnc2-GrAnc3 and D186H substitution in GrAnc7-GrAnc8 played significant roles in enhanced stability, whereas mutations near the substrate-binding site (e.g., R171A and H214S) resulted in increased PET degradation by fivefold. The H214S mutation facilitated W185 repositioning to accommodate PET chains, while its reversion (S214H) in PROSS5 reduced activity by 10-fold. Evolutionary trajectory analysis revealed an activity-stability trade-off, in which the high catalytic efficiency of *Is*PETase correlates with reduced thermostability, likely reflecting recent adaptive pressures absent in ancestral lineages. Surface charge alterations showed no functional relevance, and this emphasized the localized impact of active-site mutations. In addition, two fundamental principles were suggested in enzyme engineering: (1) PETase activity emerged recently through functional specialization from ancestral promiscuous activities; and (2) stabilizing mutations (particularly those enhancing rigidity) often compromise catalytic efficiency, necessitating balanced optimization strategies.

This work establishes ASR-rational design integration as a paradigm for enzyme engineering, which enables systematic exploration of evolutionary sequence space to bypass limitations of traditional approaches. The identification of functional mutation clusters and stability-activity trade-offs provides a roadmap for developing industrially viable biocatalysts.

#### 3.6.5 Development of the efficient biosensor for the rapid detection of enzymatic PET degradation

To advance enzyme- and microbial- mediated PET degradation, developing highly sensitive screening methods for PET-degrading enzymes is crucial. Recently, a novel fluorescent biosensor using *Comamonas thiooxidans* strain S23, a TPA-transporting and -metabolizing microorganism, was proposed (Dierkes et al., 2023). *C. thiooxidans* S23 was engineered to transport and metabolize TPA *via* a special transporter (tripartite tricarboxylate transporter, TTT) and enzymes encoded in the conserved *tphC-tphA1* operon. The catalytic enzymes are regulated by the upstream *TphR*, an *IclR*-type transcriptional regulator, and TPA can induce up to 88-fold increase in the transcription of the *tphC-tphA1* cluster. Based on these, *C. thiooxidans* S23 was genetically modified to enhance TPA detection sensitivity. Differential expression analysis identified the inducible *tphC* promoter, which was upregulated by TPA with a maximum 88.03-fold increase. Subsequently, the *sfGFP* gene was inserted between *tphR* and *tphC*, generating strain ReporTPA_UHH03. The strain showed a positive correlation between TPA concentration and fluorescence signal, with significant increases at 10 and 50 mM TPA, but no further induction at higher concentrations. To further boost sensitivity, a 3,677-bp deletion of TPA-catabolic genes (Δ*tphA2A3BA1*) was introduced into *C. thiooxidans* S23, yielding the final bioreporter ReporTPA_UHH04. Its TPA sensitivity was increased by 10,000-fold. At 1 nM TPA, it exhibited over twofold fluorescence enhancement and reached maximum response at 50 nM TPA, with no additional fluorescence increase at higher TPA concentrations. The ReporTPA_UHH04 biosensor is capable of successfully detecting enzymatic PET degradation catalyzed by various PETases, which demonstrates high sensitivity and potential for qualitative assessment of enzymatic activity on PET. It is the most sensitive bioassay for detecting TPA from PET degradation to date. With a rapid and simple 1:1 dilution method, it can analyze enzymatic supernatants within 2–4 h, which shows promise for identifying low-activity PET-degrading microorganisms and enzymes, and monitoring microbial degradation over time.

Similarly, a combined method of absorption measurements and proton nuclear magnetic resonance (^1^H NMR) analysis has also been developed for the qualitative and quantitative analysis of PET degradation products (Kornberger et al., 2024). However, to ensure measurement accuracy, further optimization of several key parameters is necessary, including solvents, pH levels, and drying techniques. Once these conditions are optimized, the absorption method can achieve sensitivity thresholds of 2.5–5 μM, and the ^1^H NMR analysis can reach 5–10 μM. This approach can significantly enhance the precision of PET degradation measurements, which is vital for plastic waste recycling and environmental conservation, thus contributing to the mitigation of the plastic waste problem.

#### 3.6.6 Rational protein design *via* loop exchange

In a recent study (Blázquez-Sánchez et al., 2023), efforts were focused on enhancing the catalytic activity of the antarctic enzyme Mors1, which could degrade PET at moderate temperatures (25°C). A chimeric design approach, specifically loop exchange (Romero and Arnold, 2009), was used to improve the activity and stability of Mors1. A chimeric enzyme (CM, chimeric Mors1) was constructed by replacing the β8-α6 loop in Mors1 with a shorter loop from LCC. This substitution led to the loss of a disulfide bridge and complete loss of enzymatic activity in CM. However, adding a cysteine residue to CM (variant CM^*A*266*C*^) to reintroduce the disulfide bridge could restore its ability to degrade polycaprolactone (PCL). Notably, CM^*A*266*C*^ had a higher optimal temperature for PET degradation (45°C) compared to Mors1 (25°C). At 45°C, CM^*A*266*C*^ was more active than Mors1, while at 25°C, its activity was lower. The PET-degrading activity of CM^*A*266*C*^ against amorphous PET films at 25°C was fivefold higher than that of wild Mors1. MD simulations of Mors1 and the CM variant suggested that the enhanced activity resulted in the changes in local flexibility within the extended loop and adjacent active-site regions, rather than the entire enzyme. For CM^*A*266*C*^, the modified loop and nearby regions showed increased movement compared to Mors1, and this extra movement was not temperature-induced, as Mors1 did not exhibit such changes at different temperatures.

This research highlights the potential of protein engineering, specifically loop exchange at the catalytic site loop, to boost the efficiency of PET-degrading enzymes. It offers a novel way to enhance enzyme performance without extensive modifications, where the increased flexibility in specific regions of the new enzyme likely contributes to its improved function. By employing more loop swaps and computational tools, it may be possible to develop even more efficient enzymes for low-temperature PET degradation. This could lead to more sustainable waste management strategies and help reduce the environmental impact of plastic pollution.

#### 3.6.7 Glutathione S-transferase used for PET-degradation under mild conditions

Recently, researchers explored the capacity of glutathione S-transferase (GST), a mammalian-origin enzyme mainly involved in detoxification, to degrade PET particles and plastic bottle debris under mild conditions (Huang et al., 2024). GST was found to efficiently degrade PET particles. Under ambient conditions with a PET-to-enzyme ratio of 22.5 kg/mg or physiological conditions at 1 atm, the degradation efficiency reached up to 95.9%, and the degrading process follows first-order kinetics with a rate of 2.6 g⋅L^–1^⋅h^–1^. When particle size decreased to less than 150 nm, the degradation efficiency climbed to 98.9%, also following first-order kinetics. However, the degradation process was inhibited by acetonitrile (ACN) and small-molecule GST inhibitors, with a significant reduction in efficiency. Additionally, factors like light irradiation, temperature, pH, and the presence of humic acid (HA) or protein would all affect the catalytic performance of GST. Notably, light irradiation was shown to be capable of enhancing the activity of GST, which revealed its adaptation to photo-oxidative stress and potential involvement in light-regulated signaling pathways.

A novel enzymatic pathway for PET depolymerization was proposed, where GST-mediated degradation predominantly involves nitration and oxidative cleavage mechanisms rather than conventional hydrolysis-based mechanisms. This conclusion was substantiated by the observed progressive enrichment of nitrogen (from 0.5% to 4.7%) and oxygen (from 18.7% to 61.6%) alongside carbon depletion (73.5%–67.7%) during degradation, coupled with characteristic spectral signatures of -C = O- and -C = N- bond formation. This process also enhanced the flexibility of the active site, which is also crucial for improved PET-degrading activity.

Besides GST, the study tested other mammalian-origin enzymes such as trypsin and CYP450 for PET degradation. Although showing potentials, the degradation efficiencies were lower than that of GST. This implies that mammalian-origin enzymes may play a role in plastic metabolism within organisms, including humans. This discovery offers a potential sustainable and eco-friendly approach to combat plastic pollution. The study not only proposes an innovative approach to plastic pollution mitigation but also advances the comprehension of plastic metabolic pathways and environmental persistence in biological systems. Furthermore, it elucidates the multifaceted mechanisms through which naturally occurring enzymatic systems degrade synthetic polymers, revealing catalytic strategies ranging from radical-mediated oxidative cleavage to processive enzymatic depolymerization.

#### 3.6.8 Synergistic strategies for PET biodegradation

Recently, a novel approach for PET biodegradation was proposed, which mimics the enzymatic mechanisms in plant cell wall hydrolysis (Taxeidis et al., 2024). This strategy centered on combining *Fusarium oxysporum* cutinase with an anionic surfactant like sodium dodecyl sulfate (SDS). The combination significantly enhanced the hydrolysis of amorphous and semi-crystalline PET, with 2.3-fold and 1.6-fold higher efficacies respectively. The improvement was ascribed to the change in surface tension, which enhanced enzyme access to the PET substrate. The use of enzyme cocktails and surfactants for PET degradation under mild conditions suggests potential for industrial-scale, and eco-friendly plastic waste management.

When cutinase was combined with ferulic acid esterases (FAEs), it achieved complete conversion of PET intermediates to TPA, and the product release was increased by up to 1.9-fold in the presence of a surfactant. The combination of cutinase and glucuronoyl esterase (GE) could also improve the degradation yields in semi-crystalline PET by up to 1.4-fold. The novel function of MtFae1, an enzyme from the CE-1 family in the CAZy database, was further verified to exhibit MHETase activity, highlighting its potential in PET degradation.

The significance of surfactants in promoting PET breakdown was also confirmed by using PEG to stabilize a *Humicola insolens* cutinase (HiC) variant for enhanced PET biodegradation (Feng et al., 2024). Under optimal conditions, adding 1% w/v PEG significantly increased the production of PET hydrolysis by-products. PEG600 was particularly effective, yielding a 64.58% increase compared to using HiC alone. In-depth studies revealed that PEG modifies the overall protein structure, enhancing catalytic performance. Additionally, PEG improved PET hydrolysis by reducing surface tension, thus increasing the interaction between HiC and PET.

#### 3.6.9 Development of switchable enzyme mimics for PET degradation

**FIGURE 14:**
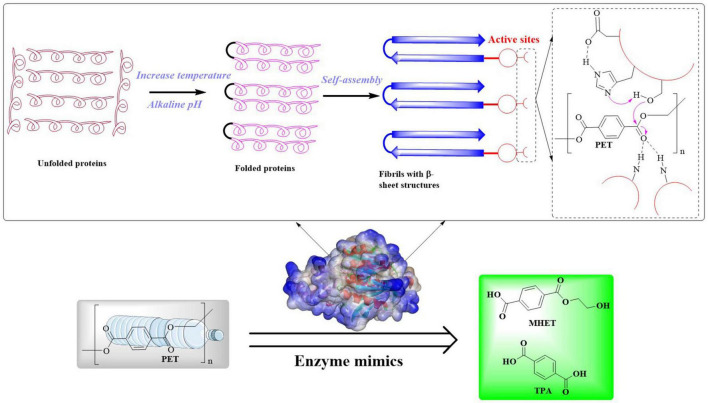
Representation of the designed peptides capable of self-assembling into enzyme-like structures capable of depolymerizing PET.

Inspired by native enzyme structures and catalytic mechanisms, recent research has successfully developed enzyme mimics to tackle PET pollution (Figure 14; Li et al., 2023c). Designed peptides capable of self-assembling into enzyme-like structures can depolymerize PET through a mechanism switchable by pH and temperature changes. In this study, two peptides (PV and PT) were designed. They incorporated the serine-histidine-aspartate active sites, similar to the catalytic triad in PETase, and were combined with the self-assembling polypeptide MAX. Purified peptides were dissolved in deionized water and added to Tris–HCl buffers at different pH values. The peptide solutions were incubated at room temperature for 24 h for self-assembly. Under varying pH and temperature conditions, these peptides underwent conformational transitions from random coil to β-sheet structures, affecting their catalytic activity.

Both PV and PT enzyme mimics are capable of effectively hydrolyzing PET, releasing MHET and TPA without BHET in the reaction mixtures. Enzymatic activities increased with the increase of pH and temperature, peaking at pH 9.0 and 65°C, and the PT peptide (∼10 μM) showed higher catalytic activity than PV (∼5 μM). pH changes likely altered amino acid charge states, which result in conformational changes in the enzyme mimics and the catalytic active site. Histidine residues were crucial in the catalytic mechanism, potentially acting as a general base to deprotonate serine residues for nucleophilic attacks. Over time, the increase in TPA and decrease in MHET indicated that PET was the initial primary substrate, and MHET becomes the main substrate as the reaction proceeded. The enzyme mimics could maintain the structural integrity after PET hydrolysis. MD simulations showed that the enzyme mimics formed fiber-like constructs rich in β-strands. Peptide PT, with a random coil and β-sheet mixed structure, had more structural flexibility and higher PET-binding affinity. Docking analysis suggested that the high catalytic activity for PET degradation result in the stable peptide-fiber formation and ordered molecular conformations, which is mainly driven by hydrogen bonding and hydrophobic interactions.

These results highlight that the switchable enzyme mimics hold promise as materials for degrading PET and reducing environmental pollution.

#### 3.6.10 Integrated chemical-biological upcycling of PET waste through engineered *Rhodococcus jostii*

To establish a sustainable solution for PET waste management, an innovative chemical-biological hybrid platform was developed coupling alkaline depolymerization with microbial valorization (Diao et al., 2023). The strategy centers on an engineered strain of *R. jostii* PET (RPET) capable of simultaneously metabolizing both TPA and EG monomers derived from PET hydrolysis, while converting these substrates into high-value lycopene. The engineering process involved a systematic multi-step approach: (1) *in silico* identification of carotenoid pathway components through whole-genome sequencing; (2) implementation of arabinose-inducible expression system (*P*_*BAD*_ promoter) to regulate the *crtEBI* operon enhancing carbon flux from isopentenyl pyrophosphate (IPP) and dimethylallyl pyrophosphate (DMAPP) precursors; (3) targeted knockout of lycopene β-cyclase gene (*crtL-*β) to prevent carotenoid diversification; (4) combinatorial overexpression of methylerythritol phosphate (MEP) pathway genes (*dxs* and *idi*) to amplify precursor supply; and (5) construction of synthetic operons for coordinated expression of rate-limiting enzymes. Notably, alkaline pretreatment achieved 98.7% PET depolymerization efficiency (w/w) within 6 h at 80°C, which generated hydrolyzates containing 12.4 g/L TPA and 6.8 g/L EG. When cultivated in fed-batch bioreactors using PET hydrolyzate as the sole carbon source, the engineered RPET strain demonstrated remarkable lycopene productivity, reaching 1.32 ± 0.15 g/L – a 523-fold enhancement compared to wild-type controls. This bioconversion efficiency represents a significant advance over previous enzymatic PET recycling systems, which typically focus on monomer recovery rather than value-added chemical synthesis (Li A. et al., 2024).

These findings establish a proof-of-concept for closed-loop PET valorization, where plastic waste is transformed into commercially relevant antioxidants through synergistic chemical and biological processing. Its unique capacity to utilize both PET-derived monomers while tolerating osmotic stress positions it as a promising chassis for developing circular economy solutions in plastic waste management. However, it should be also noted that the engineered strains (e.g., *R. jostii* RPET) expressing heterologous pathways for TPA/EG metabolism and value-added product synthesis could inadvertently transfer genetic constructs to environmental microbes, potentially disrupting ecosystems. While prevailing research prioritizes functional enhancements in microbial consortia-such as promoter optimization and chassis stabilization-critical biosafety dimensions remain underexplored. This includes systematic implementation of containment mechanisms (e.g., auxotrophic dependencies and CRISPR-based kill switches) and ecological monitoring protocols to track genetic stability post-deployment, which must be rigorously embedded within consortium engineering frameworks. Future work should prioritize risk-mitigation frameworks, such as orthogonal genetic systems or CRISPR-based biocontainment, to align the ecological promise of consortia with biosafety imperatives.

### 3.7 Synergistic microbial-enzymatic degradation of PET waste *via* a novel lipase-enzyme consortium

**FIGURE 15:**
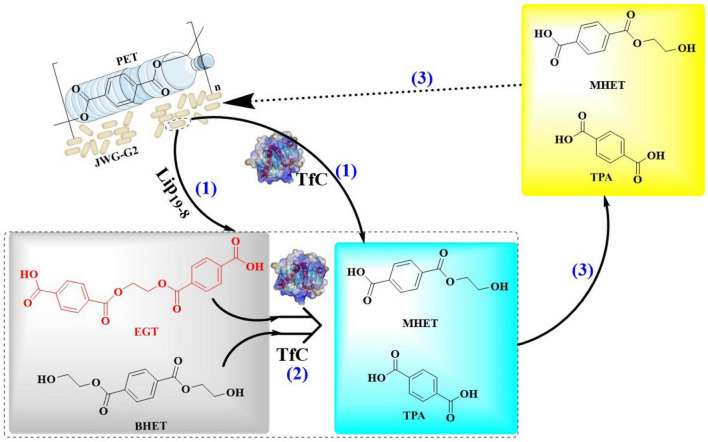
The estimated synergistic degradation pathway of microbe-enzyme system.

Recently, an innovative biocatalytic platform combining *Microbacterium oleivorans* JWG-G2 with *T. fusca* cutinase (TfC) was established to synergistically degrade BHET oligomers and high-crystallinity PET films (Figure 15; Yan et al., 2021). Notably, PET exposure induced a 1.6-fold increase in *M. oleivorans* cell density accompanied by 60% elevation in extracellular esterase activity (*p* < 0.01), which suggested the substrate-responsive secretion of hydrolytic enzymes. Crucially, a previously unreported PET degradation intermediate, ethylene glycol terephthalate (EGT), was identified during microbial treatment. EGT exhibited inhibitory effects analogous to MHET in enzymatic hydrolysis systems (Barth et al., 2015a) necessitating combinatorial biocatalyst design.

The engineered consortium achieved remarkable degradation efficiencies through sequential action: (1) co-treatment enhanced BHET oligomer degradation by 2.79-fold and PET film depolymerization by 2.26-fold with the final products MHET (330 ± 28 nM) and TPA (47 ± 5 nM); (2) 3.2-fold increased surface roughness was revealed on PET films after combinatorial treatment, indicative of cooperative bio-abrasion and enzymatic hydrolysis; (3) 47 putative PET-degrading genes were identified in *M. oleivorans* by whole-genome sequencing, including five hydrolases; and (4) biochemical characterization highlighted Lip19-8, a lidless lipase encoded by gene 19_8, as the key biocatalyst. Structural modeling revealed an exposed catalytic triad (Ser160-His285-Asp255) within a spacious substrate-binding pocket (1.342 Å), which is capable of enabling accommodation of bulky PET oligomers. Functional studies demonstrated its capacity to sequentially hydrolyze PET into BHET, EGT, and MHET, while TfC complementarity alleviated EGT-mediated inhibition to complete TPA liberation.

This work elucidates a two-tier degradation mechanism: (1) *M. oleivorans*-secreted hydrolases initiate PET surface amorphization and primary depolymerization, and (2) TfC executes terminal hydrolysis of inhibitory intermediates. The microbial-enzymatic synergy outperforms conventional single-modality approaches by 208% in total monomer recovery, establishing a prototype for overcoming crystallinity barriers in plastic bio-recycling.

Recent advances in microbial-enzyme consortia have demonstrated significant improvements in PET degradation efficiency through complementary bio-physicochemical mechanisms. A representative study (Huang et al., 2022) revealed that *Stenotrophomonas pavanii* JWG-G1 pretreatment followed by TfC treatment is able to achieve 91.4% ± 2.3% PET film weight loss. This enhancement was mainly attributed to microbial surface modification to facilitate enzymatic accessibility, as a 32% increase in surface roughness and a 10.7% reduction in hydrophobicity (WCA: from 96.6° ± 2° to 86.3° ± 2°, *p* < 0.05) were quantified.

Parallel developments in thermophilic systems further validate this synergy. A consortium combining *Bacillus thermoamylovorans* JQ3 with the engineered PETase LCC^ICCG^ achieved 65.1% PET mass reduction within 48 h under optimized conditions (1.8-2.4 × 10^8^ CFU/ml microbial density; 6.25–12.5 μg/ml enzyme dosage) (Yan et al., 2024). Kinetic analysis revealed peak depolymerization activity (3.0 mg PET⋅h^–1^) and TPA productivity (2.7 mg⋅h^–1^) during 24–36 h, and the complete degradation was achieved across a broad substrate range (30–360 g/L PET) within 96 h. Notably, this system distinguishes itself from *M. oleivorans*-TfC consortia (Yan et al., 2021) by omitting intermediate oligomer degradation (e.g., EGT formation) and instead prioritizes direct TPA liberation through: (1) biofilm-mediated enzyme immobilization, where microbial colonization enhanced PETase adsorption by 4.8-fold *via* extracellular polymeric substance-driven surface adherence; and (2) metabolic coupling, wherein microbial assimilation of soluble TPA (up to 53 μM) sustained population viability (OD_600_ ≥ 0.82), establishing a self-perpetuating degradation cycle.

Combinatorial assays demonstrated that Ces39_5-LCC^ICCG^ co-treatment maximized TPA accumulation (53 μM), and it outperformed single-enzyme systems by 7.2-fold. Structural simulations revealed the expanded substrate-binding cleft of Ces39_5 (1.580 Å vs. 1.210 Å in Lip4_120), enabling efficient 2-HE (MHET)5 processing.

These systems exemplify two distinct synergistic paradigms for PET-degradation. The surface-modifying consortia (*S. pavanii* + TfC), which is capable of prioritizing physical PET amorphization to potentiate enzymatic hydrolysis. The metabolically integrated systems (*B. thermoamylovorans* + LCC^ICCG^) are able to leverage microbial catabolism to sustain enzymatic activity. While thermophilic systems offer operational stability (50–75°C tolerance), their TPA yields remain constrained [≤53 μM vs. 330 μM in mesophilic systems (Yan et al., 2021)] due to microbial substrate competition. Nevertheless, the low enzyme demand [12.5 μg/ml vs. 50 μg/ml in conventional processes (Shabbir et al., 2020)] positions them as economically viable for industrial-scale PET biorecycling. Future optimization of multi-enzyme cascades (e.g., Ces39_5-Lip4_120-LCC^ICCG^ combinations) might bridge this yield gap while maintaining process sustainability.

### 3.8 Molecular engineering of photosynthetic microorganisms

Photosynthetic microorganisms like algae, when adhering to plastic surfaces, secrete ligninolytic and exopolysaccharide enzymes crucial for plastic degradation (Barone et al., 2024). With the progress of synthetic biology, the potential of using the marine microalga *Phaeodactylum tricornutum* to degrade PET plastics has been explored (Moog et al., 2019). This involved genetically modifying *P. tricornutum* to produce and secrete PETaseR280A-FLAG (an engineered PETase from *I. sakaiensis*), which could release PETase into the surrounding medium. Degradation products, mainly TPA and MHET, were detected in the micromolar range. At 30°C, using the culture supernatant, the secreted PETase showed activity against both PET and the copolymer polyethylene terephthalate glycol (PETG). Notably, the turnover rate for low-crystallinity PETG [total products (MHET + TPA) 26.32 μM] was ∼80-fold higher than that for bottle PET [total products (MHET + TPA) 0.31 μM]. The diatom-produced PETase remained active against industrially shredded PET in a saltwater environment, even at 21°C. SEM analysis of PET treated with PETaseR280A-FLAG revealed significant surface disruptions, confirming biodegradation.

In a related study, the green microalgae *C. reinhardtii* (CC-124 and CC-503) were used to express the PET-degrading enzyme PETase for treating plastic waste (Kim et al., 2020). A codon-optimized PETase gene was inserted into a high-strength expression vector pBR9_PETase_Cre, and then was transformed into the microalgae *via* electroporation. Western blot analysis showed that CC-124 could effectively express PETase, while CC-503 had poor growth and expression. Incubating PET samples with cell lysates from the transformed CC-124 microalgae detected TPA, indicating PET hydrolysis. For PET powder from scratched bottles, a 35.17% conversion to TPA (about 9.12 mg from 30 mg of PET powder) was achieved after 4-week incubation. PET films showed no change after 2-week incubation with cell lysates but displaying holes and dents after 4 weeks, and the TPA detected by HPLC and SEM further confirmed degradation.

These results suggest that microalgae could be a potential solution for plastic pollution treatment, especially in freshwater and terrestrial settings. Using a marine microalga instead of bacteria provides a sustainable option for plastic waste management and oceanic ecosystem bioremediation. This paves the way for developing bioreactors for PET bioremediation and closed-loop recycling strategies.

## 4 Discussion and conclusion

The rapid accumulation of PET waste poses a global environmental crisis, necessitating innovative and sustainable solutions. This review systematically explores microbial-mediated PET biodegradation as a transformative strategy for plastic waste management, emphasizing advancements in enzyme engineering, metabolic pathway optimization, and synthetic biology-driven cell factory design. By integrating interdisciplinary approaches, the field would make significant strides toward establishing a circular PET economy.

While most current studies focus on lab-scale PET degradation, this review highlights critical advances supporting real-world applicability. Engineered consortia (e.g., *Pseudomonas* + *Rhodococcus*) degraded 89% of post-consumer PET microplastics in simulated marine conditions (30°C, 3.5% salinity), which clearly demonstrates the environmental resilience. Industrial scalability is also evidenced by thermostable LCC^ICCG^ variants achieving >90% PET depolymerization at 68°C within 10 h, a timeframe compatible with industrial hydrolysis reactors. Notably, cost analyses suggest PET-to-PHA upcycling *via* engineered *P. putida* could reach $1,200/ton profitability, which is driven by the reduced pretreatment and enzyme recycling. Challenges also remain in maintaining strain robustness under nutrient-limited, multipollutant scenarios, but modular chassis designs (e.g., CRISPRi-mediated metabolic insulation) show promise for field adaptability. These findings underscore the feasibility of transitioning cell factories from bioreactors to polluted ecosystems, contingent on further pilot-scale validation of stability and enzymatic turnover in heterogeneous waste streams.

In addition, novel biotechnological approaches are increasingly facilitating PET degradation through engineered enzymatic systems and optimized microbial chassis cells. Cell surface display systems are capable of enhancing enzyme stability and reusability. Immobilization strategies, such as MOF-based MC@CaZn-MOF, would improve PET film degradation *via* spatially organized enzyme complexes. Enzyme-substrate interface engineering plays important roles in improving PET accessibility for high-crystallinity substrates compared with the non-engineered enzymes. Photosynthetic consortia offer self-sustaining, solar-driven degradation, though it currently exhibits low yields compared to heterotrophic systems. CRISPR-edited microbes with streamlined TPA pathways show promise for carbon-efficient upcycling but require further optimization.

As mentioned above, despite progress have been made in the field of microbial-mediated PET biodegradation, critical challenges still remain. (1) Enzyme limitations, mainly caused by thermostability and product inhibition (e.g., MHET accumulation), might constrain the long-term efficiency. As highlighted in this study, this could be partially addressed by computational tools like ASR and machine learning-driven evolution. However, to meet the needs for industrial application, significant efforts are still required to enhance the stability and catalytic activity of PET-degrading enzymes. (2) Optimization of the metabolic bottlenecks. Especially, the competing carbon flux (e.g., TPA vs. EG utilization) and redox imbalances necessitate finer metabolic tuning. This challenge could be settled by dynamic regulation systems, especially the promoter engineering (e.g., temperature-responsive promoters) could optimize pathway coordination. (3) Industrial scalability is also a critical aspect that requires key consideration. Low enzyme production yields and high costs hinder large-scale adoption. In order to solve this difficult problem, as highlighted algal systems (e.g., *C. reinhardtii*) and SSF might present eco-friendly alternatives but require yield optimization. (4) Environmental adaptation is another thorny issue that requires in-depth exploration during the industrial application of PET biodegradation. Microbial consortia face ecological instability in non-sterile environments. Robust synthetic communities, such as the four-species *Bacillus-Pseudomonas-Rhodococcus* consortium, must balance nutrient competition and cross-feeding dynamics. While these studies emphasize the benefits of synthetic consortia, challenges such as *R. jostii* RPET remain underexplored. For instance, the mentioned-above four-species microbial consortium (*Bacillus-Pseudomonas-Rhodococcus-P. putida*) demonstrated improved PET degradation but faced growth competition in nutrient-rich media, which necessitate optimized culturing strategies (e.g., selective inorganic salt media with dual carbon sources) to stabilize interactions and suppress inhibitory effects. Such cases highlight the delicate balance required to manage nutrient competition, cross-feeding dynamics, and metabolic incompatibilities.

In conclusion, this review underscores the transformative potential of microbial and enzymatic strategies in addressing PET pollution. By leveraging cutting-edge tools, from directed evolution to synthetic consortia, researchers have demonstrated efficient PET depolymerization, monomer assimilation, and value-added product synthesis. The integration of chemical pretreatment (e.g., alkaline hydrolysis) with biological valorization exemplifies a closed-loop paradigm, reducing reliance on fossil feedstocks. Future efforts must prioritize interdisciplinary collaboration to overcome remaining technical barriers, optimize process economics, and translate laboratory innovations into real-world solutions. As the field advances, the vision of a sustainable circular plastic economy grows increasingly attainable, offering hope for mitigating one of the most pressing environmental challenges of our time.

## References

[B1] A G. K. K A. M H. K S. G D. (2020). Review on plastic wastes in marine environment - Biodegradation and biotechnological solutions. *Mar. Pollut. Bull.* 150:110733. 10.1016/j.marpolbul.2019.110733 31767203

[B2] Aer L. Jiang Q. Zhong L. Si Q. Liu X. Pan Y. (2024a). Optimization of polyethylene terephthalate biodegradation using a self-assembled multi-enzyme cascade strategy. *J. Hazard. Mater.* 476:134887. 10.1016/j.jhazmat.2024.134887 38901251

[B3] Aer L. Qin H. Wo P. Feng J. Tang L. (2024b). Signal peptide independent secretion of bifunctional dual-hydrolase to enhance the bio-depolymerization of polyethylene terephthalate. *Bioresour. Technol.* 391:129884. 10.1016/j.biortech.2023.129884 37852506

[B4] Almeida E. Carrillo Rincón A. Jackson S. Dobson A. (2019). In silico screening and heterologous expression of a polyethylene terephthalate hydrolase (PETase)-like enzyme (SM14est) with polycaprolactone (PCL)-degrading activity, from the marine sponge-derived strain *Streptomyces* sp. SM14. *Front. Microbiol.* 10:2187. 10.3389/fmicb.2019.02187 31632361 PMC6779837

[B5] Arijeniwa V. Akinsemolu A. Chukwugozie D. Onawo U. Ochulor C. Nwauzoma U. (2024). Closing the loop: A framework for tackling single-use plastic waste in the food and beverage industry through circular economy–a review. *J. Environ. Manag.* 359:120816. 10.1016/j.jenvman.2024.120816 38669876

[B6] Arnal G. Anglade J. Gavalda S. Tournier V. Chabot N. Bornscheuer U. (2023). Assessment of four engineered PET degrading enzymes considering large-scale industrial applications. *ACS Catal.* 13 13156–13166. 10.1021/acscatal.3c02922 37881793 PMC10594578

[B7] Arnling Bååth J. Jensen K. Borch K. Westh P. Kari J. (2022). Sabatier principle for rationalizing enzymatic hydrolysis of a synthetic polyester. *JACS Au* 2 1223–1231. 10.1021/jacsau.2c00204 35647598 PMC9131473

[B8] Avilan L. Lichtenstein B. König G. Zahn M. Allen M. Oliveira L. (2023). Concentration-dependent inhibition of mesophilic PETases on Poly(ethylene terephthalate) can be eliminated by enzyme engineering. *ChemSusChem* 16:e202202277. 10.1002/cssc.202202277 36811288

[B9] Barone G. Rodríguez-Seijo A. Parati M. Johnston B. Erdem E. Cernava T. (2024). Harnessing photosynthetic microorganisms for enhanced bioremediation of microplastics: A comprehensive review. *Environ. Sci. Ecotechnol.* 20:100407. 10.1016/j.ese.2024.100407 38544950 PMC10965471

[B10] Barth M. Honak A. Oeser T. Wei R. Belisário-Ferrari M. Then J. (2016). A dual enzyme system composed of a polyester hydrolase and a carboxylesterase enhances the biocatalytic degradation of polyethylene terephthalate films. *Biotechnol. J.* 11 1082–1087. 10.1002/biot.201600008 27214855

[B11] Barth M. Oeser T. Wei R. Then J. Schmidt J. Zimmermann W. (2015a). Effect of hydrolysis products on the enzymatic degradation of polyethylene terephthalate nanoparticles by a polyester hydrolase from *Thermobifida fusca*. *Biochem. Eng. J.* 93 222–228. 10.1016/j.bej.2014.10.012

[B12] Barth M. Wei R. Oeser T. Then J. Schmidt J. Wohlgemuth F. (2015b). Enzymatic hydrolysis of polyethylene terephthalate films in an ultrafiltration membrane reactor. *J. Membr. Sci.* 494 182–187. 10.1016/j.memsci.2015.07.030

[B13] Bell E. L. Smithson R. Kilbride S. (2022). Directed evolution of an efficient and thermostable PET depolymerase. *Nat. Catal.* 8:5. 10.1038/s41929-022-00821-3

[B14] Bian X. Xia G. Xin J. Jiang S. Ma K. (2024). Applications of waste polyethylene terephthalate (PET) based nanostructured materials: A review. *Chemosphere* 350:141076. 10.1016/j.chemosphere.2023.141076 38169200

[B15] Blázquez-Sánchez P. Vargas J. Furtado A. Griñen A. Leonardo D. Sculaccio S. (2023). Engineering the catalytic activity of an Antarctic PET-degrading enzyme by loop exchange. *Protein Sci.* 32:e4757. 10.1002/pro.4757 37574805 PMC10464292

[B16] Brandenberg O. Schubert O. Kruglyak L. (2022). Towards synthetic PETtrophy: Engineering *Pseudomonas putida* for concurrent polyethylene terephthalate (PET) monomer metabolism and PET hydrolase expression. *Microb. Cell Fact.* 21:119. 10.1186/s12934-022-01849-7 35717313 PMC9206389

[B17] Brinch-Pedersen W. Keller M. B. Dorau R. Paul B. Jensen K. Borch K. (2024). Discovery and surface charge engineering of fungal cutinases for enhanced activity on Poly(ethylene terephthalate). *ACS Sustain. Chem. Eng.* 12 7329–7337. 10.1021/acssuschemeng.4c00060

[B18] Cao F. Wang L. Zheng R. Guo L. Chen Y. Qian X. (2022). Research and progress of chemical depolymerization of waste PET and high-value application of its depolymerization products. *RSC Adv.* 12 31564–31576. 10.1039/d2ra06499e 36380916 PMC9632252

[B19] Cao L. Li J. Yang Z. Hu X. Wang P. A. (2023). review of synthetic biology tools in Yarrowia lipolytica. *World J. Microbiol. Biotechnol.* 39:129. 10.1007/s11274-023-03557-9 36944859

[B20] Carniel A. Valoni É Nicomedes J. Gomes A. D. C. Castro A. M. D. (2017). Lipase from *Candida antarctica* (CALB) and cutinase from *Humicola insolens* act synergistically for PET hydrolysis to terephthalic acid. *Process Biochem.* 59 84–90. 10.1016/j.procbio.2016.07.023

[B21] Carr C. de Oliveira B. Jackson S. Laport M. Clarke D. Dobson A. (2022). Identification of BgP, a cutinase-like polyesterase from a deep-sea sponge-derived *Actinobacterium*. *Front. Microbiol.* 13:888343. 10.3389/fmicb.2022.888343 35495686 PMC9039725

[B22] Chen C. C. Han X. Li X. Jiang P. Guo R. T. (2021). General features to enhance enzymatic activity of poly(ethylene terephthalate) hydrolysis. *Nat. Catal.* 4 425–430. 10.1038/s41929-021-00616-y

[B23] Chen Z. Duan R. Xiao Y. Wei Y. Zhang H. Sun X. (2022). Biodegradation of highly crystallized poly(ethylene terephthalate) through cell surface codisplay of bacterial PETase and hydrophobin. *Nat. Commun.* 13:7138. 10.1038/s41467-022-34908-z 36414665 PMC9681837

[B24] Chen Z. Wang Y. Cheng Y. Wang X. Tong S. Yang H. (2020). Efficient biodegradation of highly crystallized polyethylene terephthalate through cell surface display of bacterial PETase. *Sci. Total Environ.* 709:136138. 10.1016/j.scitotenv.2019.136138 31887523

[B25] Cho J. Kim G. Eun H. Moon C. Lee S. (2022). Designing microbial cell factories for the production of chemicals. *JACS Au* 2 1781–1799. 10.1021/jacsau.2c00344 36032533 PMC9400054

[B26] Chu J. Zhou Y. Cai Y. Wang X. Li C. Liu Q. (2022). Flow and stock accumulation of plastics in China: Patterns and drivers. *Sci. Total Environ.* 852:158513. 10.1016/j.scitotenv.2022.158513 36075419

[B27] Clark M. Tornesakis K. König G. Zahn M. Lichtenstein B. Pickford A. (2024). Understanding the catalytic efficiency of two polyester degrading enzymes: An experimental and theoretical investigation. *ACS Omega* 9 44724–44733. 10.1021/acsomega.4c06528 39524671 PMC11541480

[B28] Costa A. M. D. Lopes V. R. D. O. Vidal L. Nicaud J. M. Coelho M. A. Z. (2020). Poly(ethylene terephthalate) (PET) degradation by *Yarrowia lipolytica*: Investigations on cell growth, enzyme production and monomers consumption. *Process Biochem.* 95 81–90. 10.1016/j.procbio.2020.04.001

[B29] Cribari M. Unger M. Unarta I. Ogorek A. Huang X. Martell J. (2023). Ultrahigh-throughput directed evolution of polymer-degrading enzymes using yeast display. *J. Am. Chem. Soc.* 145 27380–27389. 10.1021/jacs.3c08291 38051911 PMC11058326

[B30] Cui Y. Chen Y. Liu X. Dong S. Tian Y. E. Qiao Y. (2021). Computational redesign of a PETase for plastic biodegradation under ambient condition by the GRAPE strategy. *ACS Catal.* 11 1340–1350. 10.1021/acscatal.0c05126

[B31] Dai L. Qu Y. Huang J. Hu Y. Hu H. Li S. (2021). Enhancing PET hydrolytic enzyme activity by fusion of the cellulose-binding domain of cellobiohydrolase I from *Trichoderma reesei*. *J. Biotechnol.* 334 47–50. 10.1016/j.jbiotec.2021.05.006 34044062

[B32] Di Rocco G. Taunt H. Berto M. Jackson H. Piccinini D. Carletti A. (2023). A PETase enzyme synthesised in the chloroplast of the microalga *Chlamydomonas reinhardtii* is active against post-consumer plastics. *Sci. Rep.* 13:10028. 10.1038/s41598-023-37227-5 37340047 PMC10282039

[B33] Diao J. Hu Y. Tian Y. Carr R. Moon T. (2023). Upcycling of poly(ethylene terephthalate) to produce high-value bio-products. *Cell Rep.* 42:111908. 10.1016/j.celrep.2022.111908 36640302

[B34] Diao J. Tian Y. Hu Y. Moon T. (2024). Producing multiple chemicals through biological upcycling of waste poly(ethylene terephthalate). *Trends Biotechnol.* 43 620–646. 10.1016/j.tibtech.2024.10.018 39581772

[B35] Dierkes R. Wypych A. Pérez-García P. Danso D. Chow J. Streit W. (2023). An ultra-sensitive *Comamonas thiooxidans* biosensor for the rapid detection of enzymatic polyethylene terephthalate (PET) degradation. *Appl. Environ. Microbiol.* 89:e0160322. 10.1128/aem.01603-22 36507653 PMC9888244

[B36] Dissanayake L. Jayakody L. (2021). Engineering microbes to bio-upcycle polyethylene terephthalate. *Front. Bioeng. Biotechnol.* 9:656465. 10.3389/fbioe.2021.656465 34124018 PMC8193722

[B37] Ducret V. Perron K. Valentini M. (2022). Role of two-component system networks in *Pseudomonas aeruginosa* pathogenesis. *Adv. Exp. Med. Biol.* 1386 371–395. 10.1007/978-3-031-08491-1_14 36258080

[B38] Eberl A. Heumann S. Brückner T. Araujo R. Cavaco-Paulo A. Kaufmann F. (2009). Enzymatic surface hydrolysis of poly(ethylene terephthalate) and bis(benzoyloxyethyl) terephthalate by lipase and cutinase in the presence of surface active molecules. *J. Biotechnol.* 143 207–212. 10.1016/j.jbiotec.2009.07.008 19616594

[B39] Feng J. Li H. Lu Y. Li R. Cavaco-Paulo A. Fu J. (2024). Non-ionic surfactant PEG: Enhanced cutinase-catalyzed hydrolysis of polyethylene terephthalate. *Int. J. Biol. Macromol.* 273: 133049. 10.1016/j.ijbiomac.2024.133049 38857727

[B40] Francis H. Jin I. Seongjoon J. Seo H. Sagong H. Choi Y. (2019). Rational protein engineering of thermo-stable PETase from *Ideonella sakaiensis* for highly efficient PET degradation. *ACS Catal.* 9 3519–3526. 10.1021/acscatal.9b00568

[B41] Franden M. Jayakody L. Li W. Wagner N. Cleveland N. Michener W. (2018). Engineering *Pseudomonas putida* KT2440 for efficient ethylene glycol utilization. *Metab. Eng.* 48 197–207. 10.1016/j.ymben.2018.06.003 29885475

[B42] Fukuhara Y. Kasai D. Katayama Y. Fukuda M. Masai E. (2008). Enzymatic properties of terephthalate 1,2-dioxygenase of *Comamonas* sp. strain E6. *Biosci. Biotechnol. Biochem.* 72 2335–2341. 10.1271/bbb.80236 18776687

[B43] Furukawa M. Kawakami N. Tomizawa A. Miyamoto K. (2019). Efficient degradation of Poly(ethylene terephthalate) with *Thermobifida fusca* Cutinase exhibiting improved catalytic activity generated using mutagenesis and additive-based approaches. *Sci. Rep.* 9:16038. 10.1038/s41598-019-52379-z 31690819 PMC6831586

[B44] Gao R. Pan H. Kai L. Han K. Lian J. (2022). Microbial degradation and valorization of poly(ethylene terephthalate) (PET) monomers. *World J. Microbiol. Biotechnol.* 38:89. 10.1007/s11274-022-03270-z 35426614

[B45] Gercke D. Furtmann C. Tozakidis I. E. P. Jose J. (2021). Highly crystalline post consumer PET waste hydrolysis by surface displayed PETase using a bacterial whole cell biocatalyst. *ChemCatChem* 13 3479–3489. 10.1002/cctc.202100443

[B46] Goldenzweig A. Goldsmith M. Hill S. Gertman O. Laurino P. Ashani Y. (2016). Automated structure- and sequence-based design of proteins for high bacterial expression and stability. *Mol. Cell* 63 337–346. 10.1016/j.molcel.2016.06.012 27425410 PMC4961223

[B47] Groseclose T. Kober E. Clark M. Moore B. Banerjee S. Bemmer V. (2024). A high-throughput screening platform for engineering Poly(ethylene Terephthalate) hydrolases. *ACS Catal.* 14 14622–14638. 10.1021/acscatal.4c04321 39386920 PMC11459431

[B48] Guzik U. Hupert-Kocurek K. Sitnik M. Wojcieszyńska D. (2014). Protocatechuate 3,4-dioxygenase: A wide substrate specificity enzyme isolated from *Stenotrophomonas maltophilia* KB2 as a useful tool in aromatic acid biodegradation. *J. Mol. Microbiol. Biotechnol.* 24 150–160. 10.1159/000362791 24970342

[B49] Han C. Wang Q. Sun Y. Yang R. Liu M. Wang S. (2020). Improvement of the catalytic activity and thermostability of a hyperthermostable endoglucanase by optimizing N-glycosylation sites. *Biotechnol. Biofuels* 13:30. 10.1186/s13068-020-1668-4 32127917 PMC7045587

[B50] Hellesnes K. Vijayaraj S. Fojan P. Petersen E. Courtade G. (2023). Biochemical characterization and NMR Study of a PET-hydrolyzing Cutinase from *Fusarium solani pisi*. *Biochemistry* 62 1369–1375. 10.1021/acs.biochem.2c00619 36967526 PMC10116592

[B51] Herrero Acero E. Ribitsch D. Steinkellner G. Gruber K. Greimel K. Eiteljoerg I. (2011). Enzymatic surface hydrolysis of PET: Effect of structural diversity on kinetic properties of cutinases from thermobifida. *Macromolecules* 44 4632–4640. 10.1021/ma200949p

[B52] Heyde S. Arnling Bååth J. Westh P. Nørholm M. Jensen K. (2021). Surface display as a functional screening platform for detecting enzymes active on PET. *Microb. Cell Fact.* 20:93. 10.1186/s12934-021-01582-7 33933097 PMC8088578

[B53] Hong H. Ki D. Seo H. Park J. Jang J. Kim K. (2023). Discovery and rational engineering of PET hydrolase with both mesophilic and thermophilic PET hydrolase properties. *Nat. Commun.* 14:4556. 10.1038/s41467-023-40233-w 37507390 PMC10382486

[B54] Hong R. Sun Y. Su L. Gu L. Wang F. Wu J. (2019). High-level expression of Humicola insolens cutinase in *Pichia pastoris* without carbon starvation and its use in cotton fabric bioscouring. *J. Biotechnol.* 304 10–15. 10.1016/j.jbiotec.2019.07.011 31400343

[B55] Hosseinpour M. Asadi M. Eliato T. R. Vossoughi M. Alemzadeh I. (2016). Ethylene glycol biodegradation in microbial fuel cell. *Energy Sour.* 38 1096–1102. 10.1080/15567036.2013.831144

[B56] Howard S. McCarthy R. (2023). Modulating biofilm can potentiate activity of novel plastic-degrading enzymes. *NPJ Biofilms Microb.* 9:72. 10.1038/s41522-023-00440-1 37788986 PMC10547765

[B57] Howard S. Carr C. Sbahtu H. Onwukwe U. López M. Dobson A. (2023). Enrichment of native plastic-associated biofilm communities to enhance polyester degrading activity. *Environ. Microbiol.* 25 2698–2718. 10.1111/1462-2920.16466 37515381 PMC10947123

[B58] Hu J. Chen Y. (2023). Constructing *Escherichia coli* co-display systems for biodegradation of polyethylene terephthalate. *Bioresour. Bioprocess.* 10:91. 10.1186/s40643-023-00711-x 38647917 PMC10992762

[B59] Hu J. Chen X. Zhang L. Zhou J. Xu G. Ni Y. (2023). Engineering the thermostability of a d-carbamoylase based on ancestral sequence reconstruction for the efficient synthesis of d-tryptophan. *J. Agric. Food Chem.* 71 660–670. 10.1021/acs.jafc.2c07781 36541894

[B60] Huang C. Hsu J. Chung P. Cheng W. Jiang Y. Ju Y. (2013). Site-specific N-glycosylation of caprine lysostaphin restricts its bacteriolytic activity toward *Staphylococcus aureus*. *Anim. Biotechnol.* 24 129–147. 10.1080/10495398.2012.760469 23534959

[B61] Huang Q. Yan Z. Chen X. Du Y. Li J. Liu Z. (2022). Accelerated biodegradation of polyethylene terephthalate by *Thermobifida fusca* cutinase mediated by *Stenotrophomonas pavanii*. *Sci. Total Environ.* 808:152107. 10.1016/j.scitotenv.2021.152107 34864034

[B62] Huang X. Li Y. Shu Z. Huang L. Liu Q. Jiang G. (2024). High-efficiency degradation of PET plastics by glutathione S-transferase under mild conditions. *Environ. Sci. Technol.* 58 13358–13369. 10.1021/acs.est.4c02132 39012182

[B63] Hussain M. Mohsin M. Zaman W. Yu J. Zhao X. Wei Y. (2022). Multiscale engineering of microbial cell factories: A step forward towards sustainable natural products industry. *Synth. Syst. Biotechnol.* 7 586–601. 10.1016/j.synbio.2021.12.012 35155840 PMC8816652

[B64] Jaiswal S. Sharma B. Shukla P. (2019). Integrated approaches in microbial degradation of plastics. *Environ. Technol. Innov.* 17:100567. 10.1016/j.eti.2019.100567

[B65] Janek T. Mirończuk A. Rymowicz W. Dobrowolski A. (2020). High-yield expression of extracellular lipase from *Yarrowia lipolytica* and its interactions with lipopeptide biosurfactants: A biophysical approach. *Arch. Biochem. Biophys.* 689:108475. 10.1016/j.abb.2020.108475 32585312

[B66] Joho Y. Vongsouthi V. Spence M. Ton J. Gomez C. Tan L. (2023). ancestral sequence reconstruction identifies structural changes underlying the evolution of *Ideonella sakaiensis* PETase and variants with improved stability and activity. *Biochemistry* 62 437–450. 10.1021/acs.biochem.2c00323 35951410

[B67] Kawai F. Kawabata T. Oda M. (2019). Current knowledge on enzymatic PET degradation and its possible application to waste stream management and other fields. *Appl. Microbiol. Biotechnol.* 103 4253–4268. 10.1007/s00253-019-09717-y 30957199 PMC6505623

[B68] Kawai F. Oda M. Tamashiro T. Waku T. Tanaka N. Yamamoto M. (2014). A novel Ca^2 +^-activated, thermostabilized polyesterase capable of hydrolyzing polyethylene terephthalate from *Saccharomonospora viridis* AHK190. *Appl. Microbiol. Biotechnol.* 98 10053–10064. 10.1007/s00253-014-5860-y 24929560

[B69] Kim J. Park S. Tran Q. Cho D. Choi D. Lee Y. (2020). Functional expression of polyethylene terephthalate-degrading enzyme (PETase) in green microalgae. *Microb. Cell Fact.* 19:97. 10.1186/s12934-020-01355-8 32345276 PMC7189453

[B70] Kornberger D. Paatsch T. Schmidt M. Salat U. (2024). New combined absorption/1H NMR method for qualitative and quantitative analysis of PET degradation products. *Environ. Sci. Pollut. Res. Int.* 31 20689–20697. 10.1007/s11356-024-32481-0 38393574 PMC10927764

[B71] Kosiorowska K. Moreno A. Iglesias R. Leluk K. Mirończuk A. (2022b). Production of PETase by engineered *Yarrowia lipolytica* for efficient poly(ethylene terephthalate) biodegradation. *Sci. Total Environ.* 846:157358. 10.1016/j.scitotenv.2022.157358 35850328

[B72] Kosiorowska K. Biniarz P. Dobrowolski A. Leluk K. Mirończuk A. (2022a). Metabolic engineering of *Yarrowia lipolytica* for poly(ethylene terephthalate) degradation. *Sci. Total Environ.* 831:154841. 10.1016/j.scitotenv.2022.154841 35358523

[B73] Kushwaha A. Goswami L. Singhvi M. Kim B. S. (2022). Biodegradation of Poly(ethylene terephthalate): Mechanistic insights, advances, and future innovative strategies. *Chem. Eng. J.* 457:141230. 10.1016/j.cej.2022.141230

[B74] Li Y. Chen L. Zhou N. Chen Y. Ling Z. Xiang P. (2024). Microplastics in the human body: A comprehensive review of exposure, distribution, migration mechanisms, and toxicity. *Sci. Total Environ.* 946:174215. 10.1016/j.scitotenv.2024.174215 38914339

[B75] Li A. Wu L. Cui H. Song Y. Zhang X. Li X. (2024). Unlocking a sustainable future for plastics: A chemical-enzymatic pathway for efficient conversion of mixed waste to MHET and energy-saving PET recycling. *ChemSusChem* 17:e202301612. 10.1002/cssc.202301612 38385577

[B76] Li W. Jayakody L. Franden M. Wehrmann M. Daun T. Hauer B. (2019). Laboratory evolution reveals the metabolic and regulatory basis of ethylene glycol metabolism by *Pseudomonas putida* KT2440. *Environ. Microbiol.* 21 3669–3682. 10.1111/1462-2920.14703 31166064

[B77] Li A. Sheng Y. Cui H. Wang M. Wu L. Song Y. (2023a). Discovery and mechanism-guided engineering of BHET hydrolases for improved PET recycling and upcycling. *Nat. Commun.* 14:4169. 10.1038/s41467-023-39929-w 37443360 PMC10344914

[B78] Li X. Shi B. Huang J. Zeng Z. Yang Y. Zhang L. (2023b). Functional tailoring of a PET hydrolytic enzyme expressed in *Pichia pastoris*. *Bioresour. Bioprocess.* 10:26. 10.1186/s40643-023-00648-1 38647782 PMC10991172

[B79] Li X. Zhou Y. Lu Z. Shan R. Sun D. Li J. (2023c). Switchable enzyme mimics based on self-assembled peptides for polyethylene terephthalate degradation. *J. Colloid Interface Sci.* 646 198–208. 10.1016/j.jcis.2023.05.017 37196493

[B80] Liu F. Wang T. Yang W. Zhang Y. Gong Y. Fan X. (2023). Current advances in the structural biology and molecular engineering of PETase. *Front. Bioeng. Biotechnol.* 11:1263996. 10.3389/fbioe.2023.1263996 37795175 PMC10546322

[B81] Liu P. Zheng Y. Yuan Y. Han Y. Su T. Qi Q. (2023). Upcycling of PET oligomers from chemical recycling processes to PHA by microbial co-cultivation. *Waste Manag.* 172 51–59. 10.1016/j.wasman.2023.08.048 37714010

[B82] Liu P. Zhang T. Zheng Y. Li Q. Su T. Qi Q. (2021). Potential one-step strategy for PET degradation and PHB biosynthesis through co-cultivation of two engineered microorganisms. *Eng. Microbiol.* 1:100003. 10.1016/j.engmic.2021.100003 39629164 PMC11610943

[B83] Liu P. Zheng Y. Yuan Y. Zhang T. Li Q. Liang Q. (2022). Valorization of polyethylene terephthalate to muconic acid by engineering *Pseudomonas Putida*. *Int. J. Mol. Sci.* 23:10997. 10.3390/ijms231910997 36232310 PMC9569715

[B84] Liu Y. Liu Z. Guo X. Tong K. Niu Y. Shen Z. (2024). Enhanced degradation activity of PET plastics by fusion protein of anchor peptide LCI and *Thermobifida fusca* cutinase. *Enzyme Microb. Technol.* 184:110562. 10.1016/j.enzmictec.2024.110562 39653629

[B85] Liu W. Qiang K. Ye P. Li X. Zhao X. Hong J. (2024). Insight into in situ enzymatic transesterification modification of polyethylene terephthalate fibers with polyethylene glycol. *Process Biochem.* 146 140–146. 10.1016/j.procbio.2024.07.031

[B86] Liu S. Xu L. Sun Y. Yuan L. Xu H. Song X. (2024). Progress in the metabolic engineering of *Yarrowia lipolytica* for the synthesis of terpenes. *Biodes Res.* 6:0051. 10.34133/bdr.0051 39534575 PMC11555184

[B87] Lu H. Diaz D. Czarnecki N. Zhu C. Kim W. Shroff R. (2022). Machine learning-aided engineering of hydrolases for PET depolymerization. *Nature* 604 662–667. 10.1038/s41586-022-04599-z 35478237

[B88] MacLeod M. Arp H. Tekman M. Jahnke A. (2021). The global threat from plastic pollution. *Science* 373 61–65. 10.1126/science.abg5433 34210878

[B89] Mahler A. Lemming M. Jaime-Azuara A. Pedersen T. Hinge M. (2025). Chemical recycling of polymer contaminated poly(ethylene terephthalate) by neutral hydrolysis. *Waste Manag.* 192 12–19. 10.1016/j.wasman.2024.11.028 39579460

[B90] Makryniotis K. Nikolaivits E. Gkountela C. Vouyiouka S. Topakas E. (2023). Discovery of a polyesterase from *Deinococcus maricopensis* and comparison to the benchmark LCCICCG suggests high potential for semi-crystalline post-consumer PET degradation. *J. Hazard. Mater.* 455:131574. 10.1016/j.jhazmat.2023.131574 37150100

[B91] Meier A. Worch S. Böer E. Hartmann A. Mascher M. Marzec M. (2017). Agdc1p - a gallic acid decarboxylase involved in the degradation of tannic acid in the yeast blastobotrys (Arxula) adeninivorans. *Front. Microbiol.* 8:1777. 10.3389/fmicb.2017.01777 28966611 PMC5605622

[B92] Meier A. Worch S. Hartmann A. Marzec M. Mock H. Bode R. (2022). Characterization of Catechol-1,2-dioxygenase (Acdo1p) from blastobotrys raffinosifermentans and investigation of its role in the catabolism of aromatic compounds. *Front. Microbiol.* 13:872298. 10.3389/fmicb.2022.872298 35722288 PMC9204233

[B93] Meyer-Cifuentes I. Öztürk B. (2021). Mle046 Is a Marine mesophilic MHETase-like enzyme. *Front. Microbiol.* 12:693985. 10.3389/fmicb.2021.693985 34381429 PMC8351946

[B94] Meyer-Cifuentes I. Werner J. Jehmlich N. Will S. Neumann-Schaal M. Öztürk B. (2020). Synergistic biodegradation of aromatic-aliphatic copolyester plastic by a marine microbial consortium. *Nat. Commun.* 11:5790. 10.1038/s41467-020-19583-2 33188179 PMC7666164

[B95] Moog D. Schmitt J. Senger J. Zarzycki J. Rexer K. Linne U. (2019). Using a marine microalga as a chassis for polyethylene terephthalate (PET) degradation. *Microb. Cell Fact.* 18:171. 10.1186/s12934-019-1220-z 31601227 PMC6786278

[B96] Moog D. Zarzycki J. Rexer K. Erb T. Maier U. (2021). Engineering microalgae as a whole cell catalyst for PET degradation. *Methods Enzymol.* 648 435–455. 10.1016/bs.mie.2020.12.023 33579415

[B97] Mrigwani A. Pitaliya M. Kaur H. Kasilingam B. Thakur B. Guptasarma P. (2023). Rational mutagenesis of *Thermobifida fusca* cutinase to modulate the enzymatic degradation of polyethylene terephthalate. *Biotechnol. Bioeng.* 120 674–686. 10.1002/bit.28305 36514261

[B98] Mubayi V. Ahern C. Calusinska M. O’Malley M. (2024). Toward a circular bioeconomy: Designing microbes and polymers for biodegradation. *ACS Synth. Biol.* 13 1978–1993. 10.1021/acssynbio.4c00077 38918080 PMC11264326

[B99] Mückschel B. Simon O. Klebensberger J. Graf N. Rosche B. Altenbuchner J. (2012). Ethylene glycol metabolism by *Pseudomonas putida*. *Appl. Environ. Microbiol.* 78 8531–8539. 10.1128/AEM.02062-12 23023748 PMC3502918

[B100] Mudondo J. Lee H. Jeong Y. Kim T. Kim S. Sung B. (2023). Recent advances in the chemobiological upcycling of polyethylene terephthalate (PET) into value-added chemicals. *J. Microbiol. Biotechnol.* 33 1–14. 10.4014/jmb.2208.08048 36451300 PMC9895998

[B101] Nakamura A. Kobayashi N. Koga N. Iino R. (2021). Positive charge introduction on the surface of thermostabilized PET hydrolase facilitates pet binding and degradation. *ACS Catal.* 11 8550–8564. 10.1021/acscatal.1c01204

[B102] Navas L. Dexter G. Liu J. Levy-Booth D. Cho M. Jang S. (2021). Bacterial transformation of aromatic monomers in softwood black liquor. *Front. Microbiol.* 12:735000. 10.3389/fmicb.2021.735000 34566938 PMC8461187

[B103] Nikolaivits E. Taxeidis G. Gkountela C. Vouyiouka S. Maslak V. Nikodinovic-Runic J. (2022). A polyesterase from the Antarctic bacterium *Moraxella* sp. degrades highly crystalline synthetic polymers. *J. Hazard. Mater.* 434:128900. 10.1016/j.jhazmat.2022.128900 35452981

[B104] Pardo I. Jha R. Bermel R. Bratti F. Gaddis M. McIntyre E. (2020). Gene amplification, laboratory evolution, and biosensor screening reveal MucK as a terephthalic acid transporter in *Acinetobacter baylyi* ADP1. *Metab. Eng.* 62 260–274. 10.1016/j.ymben.2020.09.009 32979486

[B105] Park Y. Ledesma-Amaro R. (2023). What makes *Yarrowia lipolytica* well suited for industry? *Trends Biotechnol.* 41 242–254. 10.1016/j.tibtech.2022.07.006 35940976

[B106] Preda O. Vlasceanu A. Andreescu C. Tsatsakis A. Mezhuev Y. Negrei C. (2024). Health implications of widespread micro- and nanoplastic exposure: Environmental prevalence, mechanisms, and biological impact on humans. *Toxics* 12:730. 10.3390/toxics12100730 39453150 PMC11511527

[B107] Qi X. Yan W. Cao Z. Ding M. Yuan Y. (2021b). Current advances in the biodegradation and bioconversion of polyethylene terephthalate. *Microorganisms* 10:39. 10.3390/microorganisms10010039 35056486 PMC8779501

[B108] Qi X. Ma Y. Chang H. Li B. Ding M. Yuan Y. (2021a). Evaluation of PET degradation using artificial microbial consortia. *Front. Microbiol.* 12:778828. 10.3389/fmicb.2021.778828 35003008 PMC8733400

[B109] Qiu Y. Lei P. Wang R. Sun L. Luo Z. Li S. (2023). Kluyveromyces as promising yeast cell factories for industrial bioproduction: From bio-functional design to applications. *Biotechnol. Adv.* 64:108125. 10.1016/j.biotechadv.2023.108125 36870581

[B110] Ragaert K. Delva L. Van Geem K. (2017). Mechanical and chemical recycling of solid plastic waste. *Waste Manag.* 69 24–58. 10.1016/j.wasman.2017.07.044 28823699

[B111] Ramezani Khorsand F. Hakimi Naeini S. Molakarimi M. Dehnavi E. Zeinoddini M. Sajedi R. (2024). Surface display provides an efficient expression system for production of recombinant proteins and bacterial whole cell biosensor in *E. coli*. *Anal. Biochem.* 694:115599. 10.1016/j.ab.2024.115599 38964699

[B112] Rani A. Negi S. Fan C. Lam S. Kim H. Pan S. (2024). Revitalizing plastic wastes employing bio-circular-green economy principles for carbon neutrality. *J. Hazard. Mater.* 472:134394. 10.1016/j.jhazmat.2024.134394 38703690

[B113] Ribitsch D. Acero E. H. Greimel K. Dellacher A. Guebitz G. M. (2012). A new esterase from thermobifida halotolerans hydrolyses polyethylene terephthalate (PET) and polylactic acid (PLA). *Polymers* 4 617–619. 10.3390/polym4010617

[B114] Ribitsch D. Heumann S. Trotscha E. Herrero Acero E. Greimel K. Leber R. (2011). Hydrolysis of polyethyleneterephthalate by p-nitrobenzylesterase from *Bacillus subtilis*. *Biotechnol. Prog.* 27 951–960. 10.1002/btpr.610 21574267

[B115] Ribitsch D. Hromic A. Zitzenbacher S. Zartl B. Gamerith C. Pellis A. (2017). Small cause, large effect: Structural characterization of cutinases from *Thermobifida cellulosilytica*. *Biotechnol. Bioeng.* 114 2481–2488. 10.1002/bit.26372 28671263

[B116] Roell M. Schada von Borzykowski L. Westhoff P. Plett A. Paczia N. Claus P. (2021). A synthetic C4 shuttle via the β-hydroxyaspartate cycle in C3 plants. *Proc. Natl. Acad. Sci. U.S.A.* 118:e2022307118. 10.1073/pnas.2022307118 34001608 PMC8166194

[B117] Romano A. Varriale S. Pezzella C. Totaro G. Andanson J. Verney V. (2023). Natural deep eutectic solvents as thermostabilizer for Humicola insolens cutinase. *N. Biotechnol.* 76 118–126. 10.1016/j.nbt.2023.05.006 37257817

[B118] Romero P. Arnold F. (2009). Exploring protein fitness landscapes by directed evolution. *Nat Rev Mol Cell Biol.* 10 866–876. 10.1038/nrm2805 19935669 PMC2997618

[B119] Sadler J. Wallace S. (2021). Microbial synthesis of vanillin from waste poly(ethylene terephthalate). *Green Chem.* 23 4665–4672. 10.1039/d1gc00931a 34276250 PMC8256426

[B120] Sales J. de Castro A. Ribeiro B. Coelho M. (2021). Improved production of biocatalysts by *Yarrowia lipolytica* using natural sources of the biopolyesters cutin and suberin, and their application in hydrolysis of poly (ethylene terephthalate) (PET). *Bioprocess Biosyst. Eng.* 44 2277–2287. 10.1007/s00449-021-02603-w 34165618

[B121] Sales J. de Castro A. Ribeiro B. Coelho M. (2022). Post-consumer Poly(ethylene terephthalate) (PET) depolymerization by *Yarrowia lipolytica*: A comparison between hydrolysis using cell-free enzymatic extracts and microbial submerged cultivation. *Molecules* 27:7502. 10.3390/molecules27217502 36364329 PMC9655755

[B122] Salinas J. Martinez-Gallardo M. Jurado M. Suarez-Estrella F. Lopez-Gonzalez J. Estrella-Gonzalez M. (2025). Construction of versatile plastic-degrading microbial consortia based on ligninolytic microorganisms associated with agricultural waste composting. *Environ. Pollut.* 366:125333. 10.1016/j.envpol.2024.125333 39615570

[B123] Sánchez C. (2020). Fungal potential for the degradation of petroleum-based polymers: An overview of macro- and microplastics biodegradation. *Biotechnol. Adv.* 40:107501. 10.1016/j.biotechadv.2019.107501 31870825

[B124] Santos R. Machovsky-Capuska G. Andrades R. (2021). Plastic ingestion as an evolutionary trap: Toward a holistic understanding. *Science* 373 56–60. 10.1126/science.abh0945 34210877

[B125] Sasoh M. Masai E. Ishibashi S. Hara H. Kamimura N. Miyauchi K. (2006). Characterization of the terephthalate degradation genes of *Comamonas* sp. strain E6. *Appl. Environ. Microbiol.* 72 1825–1832. 10.1128/AEM.72.3.1825-1832.2006 16517628 PMC1393238

[B126] Schada von Borzyskowski L. Schulz-Mirbach H. Troncoso Castellanos M. Severi F. Gómez-Coronado P. A. Paczia N. (2023). Implementation of the β-hydroxyaspartate cycle increases growth performance of *Pseudomonas putida* on the PET monomer ethylene glycol. *Metab. Eng.* 76 97–109. 10.1016/j.ymben.2023.01.011 36731627

[B127] Schada von Borzyskowski L. Severi F. Krüger K. Hermann L. Gilardet A. Sippel F. (2019). Marine *Proteobacteria* metabolize glycolate via the β-hydroxyaspartate cycle. *Nature* 575 500–504. 10.1038/s41586-019-1748-4 31723261

[B128] Schneier A. Melaugh G. Sadler J. (2024). Engineered plastic-associated bacteria for biodegradation and bioremediation. *Biotechnol. Environ.* 1:7. 10.1186/s44314-024-00007-0 39026535 PMC11256910

[B129] Schwaminger S. Fehn S. Steegmüller T. Rauwolf S. Löwe H. Pflüger-Grau K. (2021). Immobilization of PETase enzymes on magnetic iron oxide nanoparticles for the decomposition of microplastic PET. *Nanoscale Adv.* 3 4395–4399. 10.1039/d1na00243k 36133462 PMC9417550

[B130] Sgro M. Chow N. Olyaei F. Arentshorst M. Geoffrion N. Ram A. (2023). Functional analysis of the protocatechuate branch of the β-ketoadipate pathway in *Aspergillus niger*. *J. Biol. Chem.* 299:105003. 10.1016/j.jbc.2023.105003 37399977 PMC10406623

[B131] Shabbir S. Faheem M. Ali N. Kerr P. Wang L. Kuppusamy S. (2020). Periphytic biofilm: An innovative approach for biodegradation of microplastics. *Sci. Total Environ.* 717:137064. 10.1016/j.scitotenv.2020.137064 32070890

[B132] Shi L. Liu P. Tan Z. Zhao W. Gao J. Gu Q. (2023). Complete depolymerization of PET wastes by an evolved PET hydrolase from directed evolution. *Angew. Chem. Int. Ed. Engl.* 62:e202218390. 10.1002/anie.202218390 36751696

[B133] Shimizu T. Inui M. (2024). Novel aspects of ethylene glycol catabolism. *Appl. Microbiol. Biotechnol.* 108:369. 10.1007/s00253-024-13179-2 38861200 PMC11166783

[B134] Sivan A. Szanto M. Pavlov V. (2006). Biofilm development of the polyethylene-degrading bacterium Rhodococcus ruber. *Appl. Microbiol. Biotechnol.* 72 346–352. 10.1007/s00253-005-0259-4 16534612

[B135] Skariyachan S. Setlur A. Naik S. Naik A. Usharani M. Vasist K. (2017). Enhanced biodegradation of low and high-density polyethylene by novel bacterial consortia formulated from plastic-contaminated cow dung under thermophilic conditions. *Environ. Sci. Pollut. Res. Int.* 24 8443–8457. 10.1007/s11356-017-8537-0 28188552

[B136] Skóra T. Popescu M. Kondrat S. (2021). Conformation-changing enzymes and macromolecular crowding. *Phys. Chem. Chem. Phys.* 23 9065–9069. 10.1039/d0cp06631a 33885078

[B137] Sleutel M. Pradhan B. Volkov A. Remaut H. (2023). Structural analysis and architectural principles of the bacterial amyloid curli. *Nat. Commun.* 14:2822. 10.1038/s41467-023-38204-2 37198180 PMC10192328

[B138] Spence E. Scott H. Dumond L. Calvo-Bado L. di Monaco S. Williamson J. (2020). The hydroxyquinol degradation pathway in *Rhodococcus jostii* RHA1 and agrobacterium species is an alternative pathway for degradation of protocatechuic acid and lignin fragments. *Appl. Environ. Microbiol.* 86 e1561–e1520. 10.1128/AEM.01561-20 32737130 PMC7499046

[B139] Sulaiman S. Yamato S. Kanaya E. Kim J. Koga Y. Takano K. (2012). Isolation of a novel cutinase homolog with polyethylene terephthalate-degrading activity from leaf-branch compost by using a metagenomic approach. *Appl. Environ. Microbiol.* 78 1556–1562. 10.1128/AEM.06725-11 22194294 PMC3294458

[B140] Taxeidis G. Nikolaivits E. Nikodinovic-Runic J. Topakas E. (2024). Mimicking the enzymatic plant cell wall hydrolysis mechanism for the degradation of polyethylene terephthalate. *Environ. Pollut.* 356:124347. 10.1016/j.envpol.2024.124347 38857840

[B141] Terauchi Y. Nagayama M. Tanaka T. Tanabe H. Yoshimi A. Nanatani K. (2022). Adsorption kinetics and self-assembled structures of aspergillus oryzae hydrophobin rola on hydrophobic and charged solid surfaces. *Appl. Environ. Microbiol.* 88:e0208721. 10.1128/AEM.02087-21 35108098 PMC8939330

[B142] Then J. Wei R. Oeser T. Barth M. Belisário-Ferrari M. Schmidt J. (2015). Ca2+ and Mg2+ binding site engineering increases the degradation of polyethylene terephthalate films by polyester hydrolases from *Thermobifida fusca*. *Biotechnol. J.* 10 592–598. 10.1002/biot.201400620 25545638

[B143] Thomson R. Carrera-Pacheco S. Gillam E. (2022). Engineering functional thermostable proteins using ancestral sequence reconstruction. *J. Biol. Chem.* 298:102435. 10.1016/j.jbc.2022.102435 36041629 PMC9525910

[B144] Tiso T. Narancic T. Wei R. Pollet E. Beagan N. Schröder K. (2021). Towards bio-upcycling of polyethylene terephthalate. *Metab. Eng.* 66 167–178. 10.1016/j.ymben.2021.03.011 33865980

[B145] Tournier V. Topham C. Gilles A. David B. Folgoas C. Moya-Leclair E. (2020). An engineered PET depolymerase to break down and recycle plastic bottles. *Nature* 580 216–219. 10.1038/s41586-020-2149-4 32269349

[B146] Valenzuela-Ortega M. Suitor J. White M. Hinchcliffe T. Wallace S. (2023). Microbial upcycling of waste PET to adipic acid. *ACS Cent. Sci.* 9 2057–2063. 10.1021/acscentsci.3c00414 38033806 PMC10683474

[B147] VanAntwerp J. Finneran P. Dolgikh B. Woldring D. (2022). Ancestral sequence reconstruction and alternate amino acid states guide protein library design for directed evolution. *Methods Mol. Biol.* 2491 75–86. 10.1007/978-1-0716-2285-8_4 35482185

[B148] Vertommen M. Nierstrasz V. Veer M. V. Warmoeskerken M. M. (2005). Enzymatic surface modification of poly(ethylene terephthalate). *J. Biotechnol.* 120 376–386. 10.1016/j.jbiotec.2005.06.015 16115695

[B149] Wang T. Yang W. Gong Y. Zhang Y. Fan X. Wang G. (2024). Molecular engineering of PETase for efficient PET biodegradation. *Ecotoxicol. Environ. Saf.* 280:116540. 10.1016/j.ecoenv.2024.116540 38833982

[B150] Wang C. Long R. Lin X. Liu W. Zhu L. Jiang L. (2024). Development and characterization of a bacterial enzyme cascade reaction system for efficient and stable PET degradation. *J. Hazard. Mater.* 472:134480. 10.1016/j.jhazmat.2024.134480 38703683

[B151] Werner A. Clare R. Mand T. Pardo I. Ramirez K. Haugen S. (2021). Tandem chemical deconstruction and biological upcycling of poly(ethylene terephthalate) to β-ketoadipic acid by *Pseudomonas putida* KT2440. *Metab. Eng.* 67 250–261. 10.1016/j.ymben.2021.07.005 34265401

[B152] Xue K. Bai Z. Fordour E. Guo S. Zhou Y. Yang Y. (2024). Bacterial surface display of PETase mutants and MHETase for an efficient dual-enzyme cascade catalysis. *Bioresour. Technol.* 408:131177. 10.1016/j.biortech.2024.131177 39097240

[B153] Yamashita T. Matsumoto T. Yamada R. Ogino H. (2024). Display of PETase on the cell surface of *Escherichia coli* using the anchor protein PgsA. *Appl. Biochem. Biotechnol.* 196 5471–5483. 10.1007/s12010-023-04837-8 38165588

[B154] Yan Z. Feng C. Zhou J. Huang Q. Chen X. Xia W. (2024). Complete degradation of PET waste using a thermophilic microbe-enzyme system. *Int. J. Biol. Macromol.* 260: 129538. 10.1016/j.ijbiomac.2024.129538 38246467

[B155] Yan Z. Wang L. Xia W. Liu Z. Gu L. Wu J. (2021). Synergistic biodegradation of poly(ethylene terephthalate) using *Microbacterium oleivorans* and *Thermobifida fusca* cutinase. *Appl. Microbiol. Biotechnol.* 105 4551–4560. 10.1007/s00253-020-11067-z 34037842

[B156] Yang J. Li Z. Xu Q. Liu W. Gao S. Qin P. (2024). Towards carbon neutrality: Sustainable recycling and upcycling strategies and mechanisms for polyethylene terephthalate via biotic/abiotic pathways. *Eco Environ. Health* 3 117–130. 10.1016/j.eehl.2024.01.010 38638172 PMC11021832

[B157] Yip A. McArthur O. Ho K. Aucoin M. Ingalls B. (2024). Degradation of polyethylene terephthalate (PET) plastics by wastewater bacteria engineered via conjugation. *Microb. Biotechnol.* 17:e70015. 10.1111/1751-7915.70015 39315602 PMC11420662

[B158] Yoshida S. Hiraga K. Takehana T. Taniguchi I. Yamaji H. Maeda Y. (2016). A bacterium that degrades and assimilates poly(ethylene terephthalate). *Science* 351 1196–1199. 10.1126/science.aad6359 26965627

[B159] Yoshida S. Hiraga K. Taniguchi I. Oda K. (2021). *Ideonella sakaiensis*, PETase, and MHETase: From identification of microbial PET degradation to enzyme characterization. *Methods Enzymol.* 648 187–205. 10.1016/bs.mie.2020.12.007 33579403

[B160] You S. Lee S. Ryu M. Song H. Kang M. Jung Y. (2023). β-Ketoadipic acid production from poly(ethylene terephthalate) waste via chemobiological upcycling. *RSC Adv.* 13 14102–14109. 10.1039/d3ra02072j 37180017 PMC10168023

[B161] Zhang J. Wang H. Luo Z. Yang Z. Zhang Z. Wang P. (2023). Computational design of highly efficient thermostable MHET hydrolases and dual enzyme system for PET recycling. *Commun. Biol.* 6:1135. 10.1038/s42003-023-05523-5 37945666 PMC10636135

[B162] Zhang X. Razanajatovo M. Du X. Wang S. Feng L. Wan S. (2023). Well-designed protein amyloid nanofibrils composites as versatile and sustainable materials for aquatic environment remediation: A review. *Eco Environ. Health* 2 264–277. 10.1016/j.eehl.2023.09.003 38435357 PMC10902511

[B163] Zhang W. Han Y. Yang F. Guan L. Lu F. Mao S. (2024). A customized self-assembled synergistic biocatalyst for plastic depolymerization. *J. Hazard. Mater.* 477:135380. 10.1016/j.jhazmat.2024.135380 39088944

[B164] Zhong-Johnson E. Dong Z. Canova C. Destro F. Cañellas M. Hoffman M. (2024). Analysis of Poly(ethylene terephthalate) degradation kinetics of evolved IsPETase variants using a surface crowding model. *J. Biol. Chem.* 300:105783. 10.1016/j.jbc.2024.105783 38395309 PMC10963241

[B165] Zhong-Johnson E. Voigt C. Sinskey A. (2021). An absorbance method for analysis of enzymatic degradation kinetics of poly(ethylene terephthalate) films. *Sci. Rep.* 11:928. 10.1038/s41598-020-79031-5 33441590 PMC7806724

[B166] Zhou X. Zhou X. Xu Z. Zhang M. Zhu H. (2024). Characterization and engineering of plastic-degrading polyesterases jmPE13 and jmPE14 from *Pseudomonas bacterium*. *Front. Bioeng. Biotechnol.* 12:1349010. 10.3389/fbioe.2024.1349010 38425995 PMC10904013

[B167] Zhu B. Ye Q. Seo Y. (2022). Enzymatic degradation of polyethylene terephthalate plastics by bacterial curli display PETase. *Environ. Sci. Technol. Lett.* 7:9. 10.1021/acs.estlett.2c00332

[B168] Zhu Y. Che R. Zong X. Wang J. Li J. Zhang C. (2024). A comprehensive review on the source, ingestion route, attachment and toxicity of microplastics/nanoplastics in human systems. *J. Environ. Manag.* 352:120039. 10.1016/j.jenvman.2024.120039 38218169

[B169] Zuo Y. Zhao M. Gou Y. Huang L. Xu Z. Lian J. (2024). Transportation engineering for enhanced production of plant natural products in microbial cell factories. *Synth. Syst. Biotechnol.* 9 742–751. 10.1016/j.synbio.2024.05.014 38974023 PMC11224930

